# Improving Route Selections in ZigBee Wireless Sensor Networks

**DOI:** 10.3390/s20010164

**Published:** 2019-12-26

**Authors:** Srikar Meka, Benedito Fonseca

**Affiliations:** 1 Underwriters Laboratories (UL) Inc., 333 Pfingsten Rd., Northbrook, IL 60062, USA; srikarmeka@gmail.com; 2 Department of Electrical Engineering, Northern Illinois University, 590 Garden Rd., DeKalb, IL 60115, USA

**Keywords:** wireless sensor networks, ZigBee, link cost estimation, routing algorithms, many-to- one routing

## Abstract

The ZigBee wireless communication specifications forecast the use of multihop routes between nodes and define that nodes select their routes based on their costs. The specifications define how to compute a route cost from the probability of successfully transmitting on each of the routes’ links; and it is recommended that such probabilities be obtained by counting received link status messages or averaging link quality indicators from received packets. In this paper, we study the performance of these two recommended procedures, show that they can lead to degraded route selections, and propose a procedure that can improve route selections without modifications to the ZigBee protocol or frame formats. Our procedure estimates the probability of successful transmission on each link, based on information from the medium access layer during unicast packet transmissions, and includes a modification into how ZigBee nodes treat routing messages internally in order to reduce variations in the link cost estimates. Focusing on a home environment with one or two hops, our simulation results show that, in several scenarios, our procedure performs better than either of the two procedures recommended in the ZigBee specifications.

## 1. Introduction

**Figure 1:**
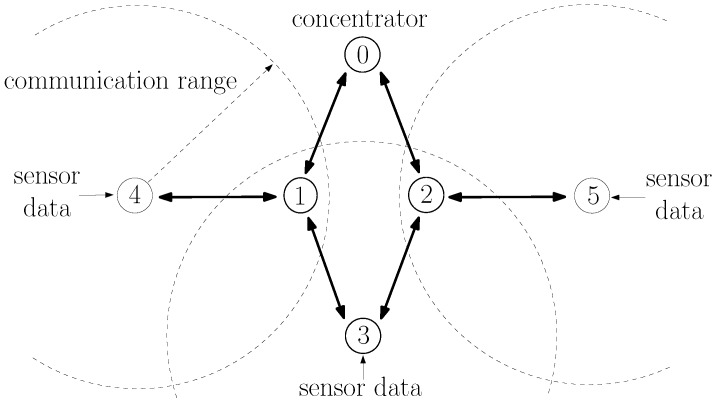
ZigBee network in which sensor data at nodes 3, 4, and 5 are transmitted to a concentrator at node 0 using nodes 1 and 2 as relays.

ZigBee is a wireless communication protocol that has been successfully used in applications ranging from home automation to industrial control and consumer electronics [1,2]. It is particularly suitable for wireless sensor networks because of the low cost of devices and their low power consumption. ZigBee operates above the IEEE 802.15.4 wireless communication standard [3], which contains medium access and physical layer procedures to enable operation in the unlicensed frequency spectrum. An additional benefit of the ZigBee protocol is its ability to extend the communication range with multihop communications [1]. Consider, for instance, the network of Figure 1. Although nodes 3, 4, and 5 are not within radio reach of node 0, they can transmit their data packets to node 1 or 2, which then relays the packets to node 0. The ZigBee protocol has detailed procedures to establish multihop routes between nodes. In fact, ZigBee contains procedures specifically tailored for many-to-one communications [4], in which several nodes transmit data to a single concentrator node, which is a typical scenario in wireless sensor networks. Although there are many products already using the ZigBee protocol, the ZigBee Alliance is still actively developing future versions [5,6], and there is still interest on ZigBee from a research perspective [7,8,9,10,11,12].

In this paper, we focus on one particular aspect that the ZigBee specifications do not fully specify: the estimation of the probability of successful transmission on a link. Such an estimation is important because it is used by nodes to select multihop routes. Often, a node has multiple candidate routes to reach another node; for example, node 3 in Figure 1 can reach node 0 through the nodes 1 or 2; and nodes are supposed to choose the route with the lowest cost. As discussed in Section 2.3, there are several ways to define the cost of a route; and ZigBee specifies the cost of a route as the sum of the cost of each link that compose a route; and the cost of each link is an inverse function of the probability of successfully transmitting on the link [13].

Although the ZigBee specifications do not specify how nodes should estimate the probability of successful transmission on a link, two possibilities are suggested (see page 338 of [13]): estimating the probability of successful transmission at the network layer by counting link status (LS) and data frames; or estimating the probability of successful transmission indirectly through the use of a physical level indicator that reflects the quality (or SINR) of a received packet. In ZigBee, such an indicator is the link quality indicator (LQI) that the IEEE 802.15.4 medium access layer (MAC) includes in every packet received and forwarded to the network layer; and the ZigBee’s network layer averages the received LQIs and maps the result into a probability of successful transmission to determine the cost of a link.

As discussed in Section 3.1, previous authors have shown that estimating link costs from beacons, such as ZigBee’s LS packets, is less accurate than estimating them from unicast data packets and have proposed alternative procedures to improve route selection [14,15,16,17,18,19,20,21,22,23,24,25,26,27,28]. Although these procedures are valuable and would improve link cost estimation and route selection, their implementation in ZigBee devices would require significant changes in the ZigBee specifications, protocol, and frame formats.

The question that motivated this paper was: can we improve link cost estimation and select better routes without changing the ZigBee protocol or frame formats? With this question in mind, our paper has two contributions:

1. We studied and compared the performances of the LS-based and LQI-based link cost estimation procedures suggested by the ZigBee specifications. We confirmed the findings of previous studies—that selecting routes based on solely the exchange of link status messages or LQI measurements can lead to poor route selections in ZigBee.
2. We proposed and evaluated the performance of a link cost estimation procedure that can be implemented without changes to the ZigBee protocol. As described in Section 6, the method that we propose is founded on estimating the probability of successful transmission by using information from the medium access control (MAC) layer regarding unicast retransmissions. Although the use of MAC layer information to estimate link costs has been considered before in other wireless networks [16,17,18,21,23,24,25,26,27,28], our method has procedures tailored to ZigBee. Furthermore, our procedure defines how to select among routes with the same cumulative costs, which is common in ZigBee links because ZigBee frame formats require the quantization of link costs into three bits. Although our procedure requires changes in the service access point between the network and MAC layers so that the MAC layer supplies more information than anticipated by the ZigBee specifications, the procedure can be implemented without changes to the ZigBee protocol or to the format of its frames. Focusing on a home environment with one or two hops, our simulations indicate that our procedure can offer better performance than either the LS-based or LQI-based procedures in several scenarios.

This paper is organized as follows: In Section 2, we provide an overview of ZigBee, the IEEE 802.15.4, the many-to-one routing protocol, and the way ZigBee specifies the computation of link and route costs. In Section 3.1, we provide more details about the previous works on estimating link costs and the LS-based and LQI-based estimation procedures suggested by the ZigBee specifications. In Section 4, we describe the simulation tool how we used it to evaluate link cost estimation procedures. Using this tool, Section 5 describes in detail, two examples that highlight the problems of the LS-based and LQI-based estimation procedures and motivated our procedure. Section 6 describes the link cost estimation method and the modified route selection procedure that we propose. In Section 7, we compare the performance of the LS-based, the LQI-based, and our proposed procedure in various topologies. Section 8 contains our conclusions and avenues for future research.

## 2. Overview of ZigBee

ZigBee is different from protocols such as IEEE 802.11, in that it specifies the use of a complete set of protocols specifically designed for device-to-device communication [1,2].

In its application layer, ZigBee defines application profiles that facilitate the communication between applications in the various devices and defines procedures for network discovery and connection establishment. ZigBee also defines the application support sub-layer (APS), which manages ongoing connections and has transport layer functionalities for end-to-end reliable data transfer with acknowledgments, retransmissions, and rejection of duplicate packets.

In its network layer, ZigBee defines procedures to establish multihop communication routes between devices, defining procedures for route discovery and routing algorithms. Four routing algorithms are defined [4]: table-based routing (similar to AODV), hierarchical tree routing, multicast routing, and many-to-one source routing. In this paper, we focus on the many-to-one (M2O) source routing algorithm because many applications of sensor networks involve many sensors transmitting information to one concentrator node. Details of the M2O algorithm are present in Section 2.2.

To support networking functions, ZigBee also defines that nodes periodically broadcast link status (LS) messages. In each LS message, a node broadcasts its current view of each neighboring link. More precisely, the LS message transmitted by a node contains a list of all neighbors of the node and contains the cost of the link from each of its neighbors to the node.

It is important to highlight that

- The link costs present in the LS message are quantized into three bits (see Section 3.4.8 of [13]).
- ZigBee differentiates between outgoing and incoming link costs; however, for M2O routing, the ZigBee specifications define that path costs be based on the maximum between them (see second paragraph of page 347 of [13]). To simplify our discussion, link costs in this paper refer to the maximum between the incoming and outgoing costs of a link.

For the medium access control (MAC) and physical layers, ZigBee specifies the use of the IEEE 802.15.4 MAC and physical layers, which are described next.

### 2.1. IEEE 802.15.4 MAC and Physical Layers

The IEEE 802.15.4 MAC layer has two operation modes: beacon and beaconless modes [3,29,30]. In this paper, we focus on the beaconless mode, which is more suitable for multihop communications. In the beaconless mode, devices associate with a coordinator device and new devices join the network by requesting beacons from devices already associated, extending the range of the network.

The IEEE 802.15.4 MAC layer controls the access of the channel through a carrier sense multiple access with collision avoidance (CSMA-CA) process. As detailed in Section 6.2.5 of [3], such a CSMA-CA procedure contains mechanisms for channel monitoring, random backoff, and retransmission.

As in other wireless protocols using CSMA-CA, ZigBee networks are prone to the hidden node problem [31,32], in which devices fail to sense each other transmissions (being hidden from each other) and may transmit at the same time, causing interference in the receiving node. For instance, in Figure 1, nodes 4 and 5 are far from nodes 0 and 3. When nodes 4 or 5 transmit, the energy received at nodes 0 and 3 is too low to trigger their carrier sense mechanism. If nodes 0 or 3 have a packet to transmit, they would then transmit at the same time as nodes 4 and 5, causing interference in the reception at nodes 1 and 2.

The IEEE 802.15.4 physical layer specifications enable operation in the unlicensed frequency spectrum at 2.4 GHz, specifying 16 channels of 2 MHz with carriers spaced by 5 MHz. As will be discussed in this paper, because WiFi IEEE 802.11 networks also operate in this band, interference and packet losses may occur in ZigBee communications when both networks operate in overlapping channels [8,33,34].

The IEEE 802.15.4, the physical layer also specifies that, whenever it sends a packet to the MAC layer, it also sends a *link quality indicator* (LQI). The LQI is a number between 0 and 255 that reflects the quality of the received symbols, being correlated with the SINR of the received packet. The LQI is also forwarded to the network layer, allowing the network layer to estimate the cost of the link, as discussed in Section 3.3.

### 2.2. Many-To-One Source Routing Algorithm

ZigBee specifies the many-to-one (M2O) Source Routing algorithm to setup routes between multiple devices and a single node, called the concentrator [1,4].

As specified in [13], to establish routes to the concentrator, the M2O routing algorithm uses a flooding of route request (RREQ) messages: the concentrator periodically broadcasts a RREQ message; nodes that receive the RREQ message rebroadcast the RREQ; and, as the RREQ travels through the network, nodes store the previous relay in their routing tables as the next hop to reach the concentrator. For example, in Figure 1, node 0 is the concentrator and periodically broadcasts a RREQ message. When nodes 1 and 2 receive the RREQ, they store in their routing tables that the concentrator can be reached directly; and, after a random delay, nodes 1 and 2 rebroadcast the RREQ message. When node 4 receives the RREQ from node 1, it stores in its routing table that node 1 is the next hop to reach node 0. Likewise, node 5 stores that node 2 is the next hop to reach node 0. The RREQ is rebroadcast by nodes until a specified maximum number of hops.

A node may have multiple routes to the concentrator, and to differentiate among routes, the RREQ message has a route cost field. This field is used by nodes to compute the cumulative route cost toward the concentrator. More precisely, whenever a node receives a RREQ message, it estimates the link cost from the transmitter to itself and adds it to the RREQ’s route cost field, forming the cumulative route cost. If the cumulative route cost is greater than the cumulative cost of a previously discovered route, then the node ignores the RREQ message. Otherwise, the routing table is updated with the next hop information and the cumulative cost; and the RREQ is rebroadcast with the route cost field updated with the cumulative cost.

The M2O algorithm allows nodes to adapt their routes to the environment. To enable routes to adapt, the concentrator broadcasts the RREQ periodically. Each broadcast of the RREQ by the concentrator marks a RREQ period. The RREQ period is defined by the RREQ identifier (RREQ-ID). The RREQ-ID is present in the RREQ message and is incremented whenever the concentrator transmits a new RREQ message. When a node receives a RREQ with a new RREQ-ID, it updates its routing table even if the cumulative route cost is higher than the cost obtained from a previous RREQ period (see first paragraph of page 348 of [13]).

The flooding of RREQ messages enables the establishment of routes from nodes to the concentrator; however, for reverse routes, the M2O algorithm uses route record (RREC) messages and source routing. A RREC message is a control packet sent by nodes to the concentrator. Whenever a node has a message to send to the concentrator, it first checks if a new RREQ period has started or if its next hop to the concentrator has changed. If either of these conditions have happened, the node sends the RREC message destined to the concentrator using the next hop node. As the RREC travels through next hop nodes, nodes append their addresses to the RREC message. When the RREC message arrives in the concentrator, it extracts and stores the whole route towards the node from the RREC message. Later, when the concentrator needs to transmit a message to the node, it uses the stored route to transmit to the target node using source routing; i.e., when transmitting the data packet, the concentrator adds the whole route in the header of the network layer. Relay nodes find out the next relay node from the header and remove their addresses before forwarding the packet.

### 2.3. Link and Route Costs

In order to enable nodes to differentiate between routes, ZigBee defines the cost of a route as follows: with $z_{1},\ldots ,z_{K}$ being the nodes that form a route, the cost of the route is defined as the sum of individual link costs:

(1) $$c_{z_{1},\ldots ,z_{K}}:=\sum_{i=1}^{K-1}c_{z_{i},z_{i+1}},$$

where $c_{z_{i},z_{i+1}}$ is the cost of the link between nodes $z_{i}$ and $z_{i+1}$.

ZigBee further defines that the cost of a link $c_{z_{1},z_{2}}$ be related to the probability of successful transmission in the link as follows: let $p_{z_{1},z_{2}}$ be the probability that a packet transmitted by $z_{1}$ is successfully received at $z_{2}$. The cost $c_{z_{1},z_{2}}$ is defined as (see Section 3.6.3.1 of [13]):

(2) $$c_{z_{1},z_{2}}:=\min\left\{7,\mathrm{round}\left(\frac{1}{{p_{z_{1},z_{2}}}^{4}}\right)\right\},$$

where the reader should note that link costs are quantized into three bits.

It should be highlighted that the probabilities of successful transmission in links, and therefore, the various link costs, vary not only because of the various distances between nodes but because of hidden node problems [31,32] and external interference [8,33,34]. For instance, node 4 in Figure 1 may generate a higher traffic load than node 5, which means that the probability that node 1 successfully receives a packet from node 3 is lower than the probability that node 2 successfully receives node 3’s packet, which means that $c_{3,1}>c_{3,2}$. Likewise, if an IEEE 802.11 WiFi network is closer to node 1 than to node 2, it may cause more interference in node 1’s reception than in node 2’s reception, causing $c_{3,1}>c_{3,2}$ as well.

Furthermore, the various link costs may vary over time. Although ZigBee networks usually involve stationary devices with a predictable traffic load, WiFi stations are mobile and their traffic load is difficult to forecast. For instance, a WiFi device may move into the area of a ZigBee network and start streaming a video of short duration, causing interference and variation in the cost of routes only during the video stream.

It is also important to note that ZigBee does not consider how the size of the packet can influence $p_{z_{1},z_{2}}$. Although different packet sizes alters $p_{z_{1},z_{2}}$, this simplification is reasonable when ZigBee is applied in applications where most application packets have approximately the same size. For instance, in a wireless sensor network where sensors behave in a similar manner and acquire the same type of measurements such a simplification is reasonable.

Lastly, we also note that ZigBee’s link cost definition does not include energy consumption. Defining link and route costs to minimize energy consumption is a common approach in the literature of route selection for wireless sensor networks [35,36,37,38,39,40,41,42,43]. Instead of addressing energy considerations in route selection, ZigBee conserves energy in battery-powered nodes by avoiding them to participate in routing procedures and by specifying power saving procedures. In this approach, battery-powered devices do not participate in routing procedures and communicate only with parent nodes, which are either the ZigBee coordinator or nodes with routing capabilities, called ZigBee Routers. Parent nodes are grid-powered and are the only nodes that participate in routing operations. Given the focus of this paper, all nodes are considered to be either the ZigBee coordinator or ZigBee Routers.

## 3. Estimating Link and Route Costs

The probabilities $p_{z_{1},z_{2}}$ needed to define the various link costs are not known a priori and nodes need to estimate them in order to estimate $c_{z_{1},z_{2}}$.

ZigBee does not specify how this estimation is to be done and implementers are supposed to specify their own estimation procedures; however, ZigBee offers two suggestions: (1) estimating $p_{z_{1},z_{2}}$ from the reception of link status (LS) or other network layer messages; and (2) estimating the cost $c_{z_{1},z_{2}}$ directly by first averaging the LQI values in packets at $z_{2}$ and then mapping it into the cost $c_{z_{1},z_{2}}$. Given that these procedures are present in the ZigBee specifications (Section 3.6.3.1 of [13]) and may be guiding implementations, we will use them as baseline for our study and we describe them in more detail after the literature review.

### 3.1. Literature Review

The first procedure suggested in the ZigBee specifications, estimating link costs from the reception of broadcast LS messages and exchanging such information in LS messages, was proposed in [28]; however, the authors in [28] used such an estimate to derive a different cost metric: the expected transmission count metric (ETX) of a link.

The second procedure suggested in the ZigBee specifications, estimating link costs from the average LQI of received packets, is similar to the MultihopLQI procedure used in the TinyOS platform. A formal reference for the MultihopLQI algorithm is no longer available; however, as reported in [44], the MultihopLQI estimates the overall cost of the route by combining the average LQI of received beacons at each node of the route. The authors in [33] have reported that link cost estimation, through LQI, performs better than procedures based on only radio signal strength, and several authors [44,45,46] have considered LQI to estimate link costs.

In contrast to beacon or LS-based procedures, and related to the method that we propose in Section 6, several authors considered MAC feedback to estimate the link costs:

- The authors in [22,24,25] relied on unicast transmissions to infer link costs; however, they focused on different link metrics based on the expected transmission time or MAC latency.
- The authors in [26] suggested the use of MAC feedback from unicast transmissions to infer link cost as opposed to relying on broadcast packets; however, as in [28], the authors in [26] focused on the estimation of the ETX metric.
- The authors in [27] proposed the EAR (efficient and accurate link-quality monitor) procedure in which nodes constantly switch between passive, cooperative, and active modes of estimation in order to estimate a metric similar to ETX.
- Focusing on the IPv6 routing protocol for low Power and lossy networks (RPL), the authors in [18] proposed that link qualities be estimated by counting the number of first time transmissions that are unsuccessful and by using an active probing mechanism where nodes send unicast messages to neighbors to estimate the link quality.

Although the procedures above explore unicast transmissions and MAC feedback to estimate links costs and select routes, they were not aimed at estimating $p_{z_{1},z_{2}}$ required by the ZigBee specification; the proposed link metrics were not evaluated considering ZigBee’s 3-bit link cost quantization; and, therefore, their route selection procedure does not consider how to select among routes with the same cumulative cost.

More recently, several authors proposed machine learning inspired procedures to estimate link costs and select routes. The authors in [14] proposed a fuzzy C-means clustering algorithm to estimate link qualities based on the packet reception rate. The authors in [15] proposed an algorithm based on reinforcement learning to control monitoring and probing mechanisms to estimate link qualities. The authors in [16] proposed an unsupervised learning technique to select network features to better classify the quality of links. The authors in [17] evaluated various machine learning algorithms that use packet reception rates, LQI, and SINR metrics as input and output the probability of successful delivery in a link. The authors in [19] proposed that nodes monitor RSSI, SINR, and packet reception rates; exchange information with neighbors; and use this information as input to a supervised learning algorithm that uses labeled training samples to estimate the quality of links. The authors in [47] proposed estimating the probability of successful packet transmission in a link by using wavelet and neural network techniques. Their approach would require decomposing measurements of the SINR into a time-varying component and a non-stationary random part. The authors in [48] proposed a hybrid online machine learning algorithm to estimate the quality of candidate links. Their approach combines current samples of link quality with baseline samples previously learned from past samples. The authors in [49] used the packet reception rates between nodes and their neighbors to select routes and propose a distributed learning automaton algorithm to choose routes that satisfy quality-of-service requirements. Although these algorithms are valuable and could improve the estimation of link costs and the selection of routes, such implementations would require nodes to implement machine learning techniques and require significant changes in the ZigBee specifications, protocol, and frame format.

### 3.2. Link Status (LS)-Based Estimation Procedure

In the LS-based estimation procedure suggested in the ZigBee specifications (Section 3.6.3.1 of [13]), the cost $c_{z_{1},z_{2}}$ of a node $z_{1}$ transmitting to a node $z_{2}$ is estimated by first estimating the probability $p_{z_{1},z_{2}}$ that $z_{1}$ successfully transmits a packet to $z_{2}$; and such an estimate is obtained from the transmission of broadcast LS messages.

To obtain the estimate ${\hat{p}}_{z_{1},z_{2}}^{\left(LS\right)}\left(t\right)$ at time *t*, let $T_{avg}$ be the duration of the estimation window; let $N_{z_{1}}^{(LS),tx}\left(t\right)$ be the number of LS messages transmitted by node $z_{1}$ in the last $T_{avg}$ seconds; let $N_{z_{1},z_{2}}^{(LS),rx}\left(t\right)$ be the number of these messages that were received by node $z_{2}$. The estimate for ${\hat{p}}_{z_{1},z_{2}}^{\left(LS\right)}\left(t\right)$ is given by

(3) $${\hat{p}}_{z_{1},z_{2}}^{\left(LS\right)}\left(t\right):=\frac{N_{z_{1},z_{2}}^{(LS),rx}\left(t\right)}{N_{z_{1}}^{(LS),tx}\left(t\right)};$$

From the estimate ${\hat{p}}_{z_{1},z_{2}}^{\left(LS\right)}\left(t\right)$, the estimate ${\hat{c}}_{z_{1},z_{2}}^{\left(LS\right)}\left(t\right)$ is obtained with

(4) $${\hat{c}}_{z_{1},z_{2}}^{\left(LS\right)}\left(t\right):=\min\left\{7,\mathrm{round}\left(\frac{1}{{{\hat{p}}_{z_{1},z_{2}}^{\left(LS\right)}\left(t\right)}^{4}}\right)\right\}.$$

It is important to observe that ${\hat{c}}_{z_{1},z_{2}}^{\left(LS\right)}\left(t\right)$ is computed at the node $z_{2}$, which is the node receiving the LS messages; but ${\hat{c}}_{z_{1},z_{2}}^{\left(LS\right)}\left(t\right)$ is used by node $z_{1}$ in routing decisions. Recall from the description of the M2O routing algorithm in Section 2.2 that a node $z_{1}$ needs to estimate $c_{z_{1},z_{2}}$ to determine the cumulative cost toward the concentrator node if it receives a RREQ from node $z_{2}$. Since $c_{z_{1},z_{2}}$ is estimated at node $z_{2}$, node $z_{2}$ needs to transmit its estimate ${\hat{c}}_{z_{1},z_{2}}^{\left(LS\right)}\left(t\right)$ to node $z_{1}$. In ZigBee, this is accomplished with LS messages as well: whenever node $z_{2}$ transmits its own LS message, it attaches to it the latest cost estimates that it computed from any of its neighbors, including ${\hat{c}}_{z_{1},z_{2}}^{\left(LS\right)}\left(t\right)$. Note further that, to compute ${\hat{c}}_{z_{1},z_{2}}^{\left(LS\right)}\left(t\right)$, node $z_{2}$ is able to determine $N_{z_{1}}^{(LS),tx}\left(t\right)$ because it follows the same procedure for transmitting LS messages, and therefore, $N_{z_{1}}^{(LS),tx}\left(t\right)\approx N_{z_{2}}^{(LS),tx}\left(t\right)$.

From the individual link costs, the cumulative cost of a route $z_{1},\ldots ,z_{K}$ is given by

(5) $${\hat{c}}_{z_{1},\ldots ,z_{K}}^{\left(LS\right)}\left(t\right)=\sum_{i=1}^{K-1}{\hat{c}}_{z_{i},z_{i+1}}^{\left(LS\right)}\left(t\right),$$

where we observe that ${\hat{c}}_{z_{1},\ldots ,z_{K}}^{\left(LS\right)}\left(t\right)$ is computed in a distributed manner. For instance, consider the route 3,1,0 in Figure 1. As described in Section 2.2, node 0 constantly estimates ${\hat{c}}_{1,0}^{\left(LS\right)}\left(t\right)$ and sends its latest estimate to node 1 whenever it sends a LS packet. Likewise, node 1 constantly estimates ${\hat{c}}_{3,1}^{\left(LS\right)}\left(t\right)$ and sends its latest estimate to node 3 whenever it sends a LS packet. Whenever a RREQ packet is broadcast over the network, node 1 adds the latest received ${\hat{c}}_{1,0}^{\left(LS\right)}\left(t\right)$ to the route cost field of the RREQ message. When node 3 receives the RREQ, it adds the latest received ${\hat{c}}_{3,1}^{\left(LS\right)}\left(t\right)$ to the cost in the route cost field, obtaining ${\hat{c}}_{3,1,0}^{\left(LS\right)}\left(t\right)$.

We further note that both the individual link costs and the route cost are functions of time because of the randomness in the reception of LS messages.

### 3.3. Link Quality Indicator (LQI)-Based Estimation Procedure

In the LQI-based estimation procedure suggested in the ZigBee specifications (Section 3.6.3.1 of [13]), the cost $c_{z_{1},z_{2}}$ of a node $z_{1}$ transmitting to a node $z_{2}$ is estimated from the average LQI of received messages: Let $T_{avg}$ be the duration of the estimation window; let $N_{LQI}$ be the number of transmissions from node $z_{1}$ successfully received at node $z_{2}$ during the last $T_{avg}$ seconds; and let $LQI_{z_{1},z_{2}}\left(j\right)$ be the value of the LQI at the $j^{th}$ received packet. The average LQI at time *t* is given by

(6) $${\bar{LQI}}_{z_{1},z_{2}}\left(t\right):=\frac{1}{N_{LQI}}\sum_{j=1}^{N_{LQI}}LQI_{z_{1},z_{2}}\left(j\right),$$

where we highlight that $N_{LQI}$ includes both broadcast and unicast received messages.

From the average LQI, the estimated cost ${\hat{c}}_{z_{1},z_{2}}^{\left(LQI\right)}\left(t\right)$ is obtained from the mapping shown in Table 1; i.e., ${\hat{c}}_{z_{1},z_{2}}^{\left(LQI\right)}\left(t\right)$ is obtained from one of seven LQI intervals corresponding to each of the seven possible costs. As the ZigBee specifications suggest (see page 338 of [13]), the LQI intervals of Table 1 should be obtained based on tests on the actual hardware. In here, the LQI intervals were obtained from off-line tests as follows: In a network of only 2 nodes without interference, the distance between the 2 nodes were varied, and at each distance, node $z_{1}$ transmitted various packets to node $z_{2}$. For each distance, the average LQI value and the ratio of successful transmissions were computed. The ratio of successful transmissions was then used as $p_{z_{1},z_{2}}$ in the link cost Equation (2) to obtain the cost for the average LQI value.

It is important to observe that ${\bar{LQI}}_{z_{1},z_{2}}\left(t\right)$ is obtained only from successfully received packets; i.e., if the SINR of a packet is so low that the IEEE 802.15.4 receiver cannot decode the packet, then the packet is discarded. No packet is sent to upper layers, and no LQI indication is generated.

As in the LS-based procedure, the cost estimate ${\hat{c}}_{z_{1},z_{2}}^{\left(LQI\right)}\left(t\right)$ is obtained at the receiving node $z_{2}$; the latest estimate is transmitted to node $z_{1}$ when node $z_{2}$ transmits its LS messages; the route cost

(7) $${\hat{c}}_{z_{1},\ldots ,z_{K}}^{\left(LQI\right)}\left(t\right)=\sum_{i=1}^{K-1}{\hat{c}}_{z_{i},z_{i+1}}^{\left(LQI\right)}\left(t\right),$$

is computed in a distributed manner, and ${\hat{c}}_{z_{1},\ldots ,z_{K}}^{\left(LQI\right)}\left(t\right)$ varies over time due to randomness in LQI measurements.

## 4. Simulation Tool to Evaluate Link Cost Estimation Procedures

In order to evaluate the LS-based, the LQI-based, and our proposed estimation procedures, we used the ns-3 simulator [50]. Ns-3 is an open-source simulator specifically designed to simulate communication protocols. It has been being actively developed for almost 10 years and has been supported by grants from DARPA and NSF.

The current version of ns-3 contains models for the IEEE 802.11 and the IEEE 802.15.4 MAC and physical layers. For this study, we implemented the required portions of the ZigBee APS and network layers, including an implementation of the M2O routing algorithm, generation of LS messages, and protocol overheads.

Ns-3 has detailed channel models to simulate wireless channels. Ns-3 manages the transmission of packets from the transmitter to any receiver, considering both noise and interference. We used ns-3’s channel and propagation model for IEEE 802.11 and IEEE 802.15.4 without changes. Details of these models can be found in [51].

### Parameters Common to All Simulations

In the upcoming sections, we describe simulations used to evaluate the performance of the LS-based, the LQI-based, and our proposed procedure in various scenarios. All of such simulations will use the configurations described on this section.

Regarding the application layer, a node $z_{i}$ that connects to a sensor (e.g., nodes 3, 4, and 5 in Figure 1) generates application messages periodically, with an interarrival time uniformly distributed between 0 and a maximum interarrival time, which we vary to generate different averages of packets generated per second. Each application message (sensor data) contains 12 bytes, which are encapsulated by an 8-byte APS header, and subsequently an 8-byte network header, before being sent to the MAC layer (total of 28 bytes). Application messages are sent using the APS reliable data transfer service; i.e., when receiving the application message, node 0’s APS layer generates an 8-byte acknowledgment (APS-ACK) frame towards the sending node. Nodes always wait for the APS-ACK message to arrive before sending a new message. If the APS-ACK message does not arrive after a timeout period of 800 ms, the APS layer retransmits the message up to three times. If the APS-ACK message has not arrived and a new message arrives, the new message is buffered. If the buffer is already full, the message is discarded.

Regarding the network layer, we considered that all ZigBee nodes were full-functioning devices able to participate in routing procedures. All ZigBee nodes generate LS messages every one second with an added random jitter uniformly distributed between 10 and 40 ms, and in all examples, we considered that node 0 was the concentrator node and nodes used the M2O routing algorithm to reach the concentrator. The concentrator was configured to send RREQ messages every 10 s; and the concentrator radius was configured such that the RREQ message was rebroadcast by at most one hop in all but the two-hop scenario of Section 7.1.3, in which the radius was configured for two hops. All the link cost estimation procedures used an averaging window of $T_{avg}=81$ s.

Regarding the IEEE 802.15.4 MAC and physical layers, all simulations used the default parameters of the ns3 model [52]. Among the MAC parameters, we highlight that the CSMA procedure was configured to retransmit packets up to three times before dropping the packet. Among the physical parameters, we highlight that transceivers required 192 microseconds to switch between receive and transmit modes, and vice-versa. All ZigBee devices were configured to transmit with 0 dBm power and operate at channel 11, centered at 2.405 GHz.

Regarding simulations with WiFi IEEE 802.11 stations, the WiFi access point was configured to have a server that transmitted an application packet of 972 bytes to each of two WiFi stations every $T_{WiFi}=972*8/R_{WiFi}$ s, where $R_{WiFi}$ s the application data throughput that we varied to generate different traffic loads. The WiFi application data was sent over UDP/IPv4. The IEEE 802.11 MAC layer exchanged RTS/CTS messages before sending the data, and we considered WiFi devices using IEEE 802.11n in Greenfield (HT) mode, with a modulation and coding scheme (MCS) level 0 with 800 ns guard spacing and occupying a 20 MHz bandwidth channel, which results in 6.5 Mbps PHY transmission rate. Considering the transmission to the two stations and the time to transmit the RTS/CTS and the MAC ACK, WiFi stations occupy the channel for at least 2.94 ms every $T_{WiFi}$ seconds, which means that the fraction of time in which WiFi transmissions occupy the channel is at least $0.00294/T_{WiFi}$. The WiFi devices transmit at 0 dBm and the 20 MHz channel is centered at 2.412 GHz, which overlaps with ZigBee transmissions centered at 2.405 GHz.

Simulations are performed for 400 s and results are collected from the last 300 s of the simulation in ZigBee-only scenarios. In scenarios where a WiFi interferer starts at time t=100 s, results are collected from the last 220 s of the simulation.

## 5. Motivating Examples

To motivate the procedure that we propose, we consider first a few examples using single simulation runs to observe the problems of both the LS-based and LQI-based procedures. Analysis considering multiple simulation runs are present in Section 7.

### 5.1. Example 1: Symmetric Topology

Consider the topology shown in Figure 1, in which sensors at node 3 at (0,−80), node 4 at (−130,0), and node 5 at (130,0) send sensor measurements to the concentrator 0 at (0,80); and node 1 at (−35,0) and node 2 at (35,0) are available for routing packets. Using the propagation model adopted for the IEEE 802.15.4 physical layer in the ns-3 simulator, nodes 4 and 5 are hidden from nodes 0 and 3.

The goal of this example was to illustrate how the LS-based and the LQI-based procedures influence the route selection of node 3, which has two routes available to reach node 0: routes 3,1,0 and 3,2,0.

Consider first a low traffic load scenario where node 3 generates an average of 0.5 of a packets and nodes 4 and 5 generate an average of 0.02 packets. Given the symmetry of the scenario, both routes have the same cost; i.e., $c_{3,1,0}=c_{3,2,0}$. We separately simulated the performance of the LS-based and the LQI-based estimation procedures and observed the estimated cumulative costs measured at node 3. In this case, both estimation procedures produced cumulative cost estimates ${\hat{c}}_{3,1,0}^{\left(LS\right)}\left(t\right)={\hat{c}}_{3,2,0}^{\left(LS\right)}\left(t\right)={\hat{c}}_{3,1,0}^{\left(LQI\right)}\left(t\right)={\hat{c}}_{3,2,0}^{\left(LQI\right)}\left(t\right)=2$ most of the times and with very little variance. The same results were obtained by simulating the system with nodes 3, 4, and 5 all generating 0.5 packets/s. This shows that, at least for symmetric topologies with low traffic load, either estimation procedure could be used and there would be no need for more elaborate procedures.

Consider now a higher traffic load scenario in which nodes 3, 4, and 5 all generate 20 packets/s. The scenario is still symmetric and both routes still have the same cost; i.e., $c_{3,1,0}=c_{3,2,0}$.

**Figure 2:**
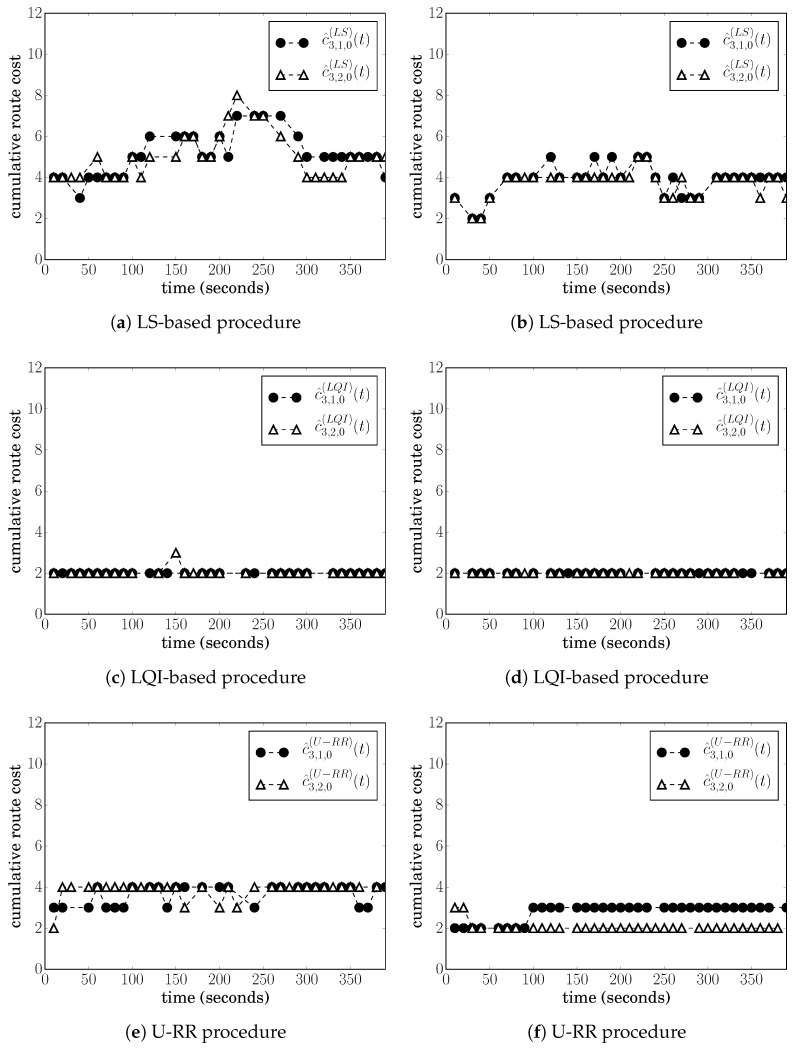
Cumulative route costs using the LS-based procedure, the LQI-based procedure, and the U-RR procedure (defined in Section 6) in topology of Figure 1. Results in (**a**,**c**,**e**) correspond to Example 1 in Section 5.1. Results in (**b**,**d**,**f**) correspond to Example 2 in Section 5.2.

Figure 2a shows the cost estimates produced over the course of a single simulation run considering the LS-based procedure; and Figure 2c shows the cost estimates produced by the LQI-based procedure. Both graphs show the cumulative cost at node 3 whenever it received a RREQ rebroadcasted from nodes 1 and 2.

From Figure 2a, we can observe that the LS-based estimated costs ${\hat{c}}_{3,1,0}^{\left(LS\right)}\left(t\right)$ and ${\hat{c}}_{3,2,0}^{\left(LS\right)}\left(t\right)$ varied significantly over the course of the simulation, even though $c_{3,1,0}=c_{3,2,0}$.

In contrast, from Figure 2c, we can observe that the LQI-based estimates were ${\hat{c}}_{3,1,0}^{\left(LS\right)}\left(t\right)={\hat{c}}_{3,2,0}^{\left(LS\right)}\left(t\right)=2$ most of the times and with very little variance.

### 5.2. Example 2: Asymmetric Topology

Consider still, the topology shown in Figure 1, but assume for this example an asymmetric traffic load: node 3 generates an average of 20 packets; node 4 generates an average of 10 packets; and node 5 generates an average of 0.5 packets.

Because node 4 generates more packets than node 5, the chance that node 1 is receiving a packet from node 4 when node 3 transmits to node 1 is higher than the chance that node 2 is receiving a packet from node 5 when node 3 transmits to node 2, which means that $c_{3,1,0}>c_{3,2,0}$ and it is desirable that node 3 chooses route 3,2,0.

Considering all other parameters as before, we simulated this topology with both the LS-based and LQI-based procedures.

For the LS-based procedure, Figure 2b shows the cumulative cost at node 3 at every RREQ received. It is possible to observe that, in some instances, the LS-based procedure was able to obtain ${\hat{c}}_{3,1,0}^{\left(LS\right)}\left(t\right)>{\hat{c}}_{3,2,0}^{\left(LS\right)}\left(t\right)$ and recognize that route 3,2,0 is preferred over 3,1,0; however, ${\hat{c}}_{3,1,0}^{\left(LS\right)}\left(t\right)={\hat{c}}_{3,2,0}^{\left(LS\right)}\left(t\right)$ most of the time. At those instances, node 3 would randomly choose between the two routes and could choose the suboptimal route 3,1,0 until the next RREQ arrives. For this particular simulation run, node 3 chose route 3,1,0 35% of the time.

For the LQI-based procedure, the cumulative costs at node 3 were similar to the costs shown in Figure 2c; i.e., both routes 3,1,0 and 3,2,0 were estimated to have the same cumulative cost 2, meaning that the LQI-based procedure was not able to recognize that route 3,1,0 had more instances of hidden node problems. Seeing both routes with the same cumulative cost, node 3 chose between routes 3,1,0 and 3,2,0 randomly. For this particular simulation run, node 3 chose route 3,1,0 64% of the time.

Sending over the suboptimal route 3,1,0 is undesirable because it increases the probability of packet losses, causing unnecessary MAC retransmissions and traffic load in the channel. For these particular simulation runs, there was an average of 108 unnecessary MAC transmissions per 1000 messages transmitted by node 3 when the LS-based procedure was used; and this number increased to 128 when the LQI-estimator was used.

### 5.3. Analyzing the LS-Based and LQI-Based Estimation Procedures

There are two main conclusions from Examples 1 and 2:

- The LS-based procedure produces estimates with higher variance than the LQI-based procedure.
- The LQI-based procedure is blind to hidden node instances.

The main reason why the LS-based cost estimates ${\hat{c}}_{3,1,0}^{\left(LS\right)}\left(t\right)$ and ${\hat{c}}_{3,2,0}^{\left(LS\right)}\left(t\right)$ had a high variance in Example 1 is because of hidden node instances. Recall that LS-based cost estimates are obtained from the ratio of received LS messages, which are sent in MAC broadcast mode, without acknowledgments or retransmissions. In Example 1, nodes 4 and 5 are hidden from node 3, which means that if node 4 or 5 transmits to node 1 or 2 while node 3 transmits its LS message, then node 1 or 2 might not be able to receive the LS message, causing a drop in the estimated ${\hat{p}}_{3,1}^{\left(LS\right)}\left(t\right)$ or ${\hat{p}}_{3,2}^{\left(LS\right)}\left(t\right)$ and an increase in the corresponding cost estimates.

It is also possible to justify the high variance of LS-based cost estimates as follows: recall that $N_{3,1}^{(LS),rx}\left(t\right)$ is the number of LS messages sent by node 3 and received by node 1; and, assuming that LS transmissions are independent, $N_{3,1}^{(LS),rx}\left(t\right)$ is a binomial random variable with parameters $N_{3}^{(LS),tx}$ and $p_{3,1}$. For the example that produced Figure 2a, the averaging window is $T_{avg}=81$ s and LS messages are sent at 1-second intervals with a random delay, which means $N_{3}^{(LS),tx}=80$. The actual $p_{3,1}$ for this example was measured as $p_{3,1}\approx 0.79$. With such a $p_{3,1}$, $N_{3,1}^{(LS),rx}\left(t\right)$ varies significantly around its mean (63.2), which means that the estimated ${\hat{p}}_{3,1}^{\left(LS\right)}\left(t\right)$ varies around 0.79, causing the estimated cost to also vary. More precisely, if node 1 receives $N_{3,1}^{(LS),rx}\left(t\right)\in \left(72,80\right]$ LS messages from node 3 during the averaging window, it estimates ${\hat{p}}_{3,1}^{\left(LS\right)}\left(t\right)\in \left(0.9,1.0\right]$, which maps into a link cost estimate of ${\hat{c}}_{3,1}^{\left(LS\right)}\left(t\right)=1$. Likewise, if $N_{3,1}^{(LS),rx}\left(t\right)\in \left(64,72\right]$, then ${\hat{c}}_{3,1}^{\left(LS\right)}\left(t\right)=2$; if $N_{3,1}^{(LS),rx}\left(t\right)\in \left(60,64\right]$, then ${\hat{c}}_{3,1}^{\left(LS\right)}\left(t\right)=3$; and if $N_{3,1}^{(LS),rx}\left(t\right)\in \left(54,60\right]$, then ${\hat{c}}_{3,1}^{\left(LS\right)}\left(t\right)=4$. With the actual $p_{3,1}\approx 0.79$, $P[N_{3,1}^{(LS),rx}\left(t\right)\in \left(72,80\right]]\approx 0.003$, $P[N_{3,1}^{(LS),rx}\left(t\right)\in \left(64,72\right]]\approx 0.367$, $P[N_{3,1}^{(LS),rx}\left(t\right)\in \left(60,64\right]]\approx 0.405$, and $P[N_{3,1}^{(LS),rx}\left(t\right)\in \left(54,60\right]]\approx 0.215$, which means that variations of ${\hat{c}}_{3,1}^{\left(LS\right)}\left(t\right)$, and therefore, the variations of ${\hat{c}}_{3,1,0}^{\left(LS\right)}\left(t\right)$ observed in Figure 2a,b are fairly common.

Variations in LS-based estimates could certainly be reduced if one increases the averaging window $T_{avg}$; however, a designer cannot increase $T_{avg}$ too much; otherwise, nodes would not able to adapt to changes in the environment. For instance, consider a WiFi device that consumes a data stream for five minutes. Such a WiFi device would cause interference in nearby ZigBee nodes and it is desirable that ZigBee nodes detect the presence of the additional interference and adjust their routes as soon as possible. If $T_{avg}$ is increased, then ZigBee nodes would take longer times to adjust their routes.

In contrast to LS-based estimates, LQI-based estimates vary much less, even in scenarios with hidden nodes. The main reason for this is the higher number of available samples in the LQI-based procedure. For instance, the LQI-based procedure at node 1 extracts a LQI sample used to estimate the ${\hat{c}}_{3,1}^{\left(LS\right)}\left(t\right)$ on every packet received from node 3, not only from LS messages. Consider Example 1: node 3 transmits an average of 20 packets/s; and, considering the averaging window of $T_{avg}=81$ s, node 1 has at least 1600 LQI samples to average, which is much more than the number of LS messages (80) transmitted in the same averaging window.

Although the LQI-based procedure performed well in Example 1, it was not able to differentiate between routes 3-1-0 and 3-2-0 in Example 2 because it is blind to hidden node problems. To understand this, recall that the LQI-based procedure estimates the cost of a link based on the average of the LQI in *received* packets. If a packet is not received, then it is not considered in the LQI average. For instance, assume node 1 is receiving a packet from node 3. Because node 3 is hidden from node 4, node 4 is unaware of node 3’s transmission and transmits at the same time, causing strong interference and possibly packet loss at node 1. If the packet is indeed lost, which is likely because of the similar distances between nodes 1 and 3 and between nodes 1 and 4, then such an event is *not* captured in the LQI average because the MAC drops the packet and no information is sent to the network layer. Later, when node 4 is no longer transmitting, node 3 retransmits the packet, which then arrives at node 1 without interference and with a high LQI. As a result, the LQI-based estimates are based on only high LQI packets that are transmitted during times of no hidden node.

It should be mentioned that, if node 4 were farther away from node 1, then the interference power would be lower; the probability of successful reception would increase; and a packet with lower LQI would be received and considered by the LQI-based procedure. However, this example shows that there are reasonable scenarios in which the LQI-based procedure would not be able to detect hidden node problems.

## 6. Proposed Link Cost Estimation and Modified Route Selection Procedure

Motivated by the problems faced by the LS-based and LQI-based procedures, we propose the use of the following modified link cost estimation and route selection procedures.

### 6.1. Link Cost Estimation Procedure

We propose that the cost of a link still be computed as in the ZigBee specification; i.e., the cost $c_{z_{1},z_{2}}$ between nodes $z_{1}$ and $z_{2}$ is still computed using the probability of successful packet transmission $p_{z_{1},z_{2}}$ in (2); however, we propose that $p_{z_{1},z_{2}}$ be estimated not only from LS packets, but also from any unicast packet transmissions from $z_{1}$ to $z_{2}$. The rationale is to increase the number of measurements and reduce the estimator variance.

To define the estimator that we propose, we first describe the ideal estimator. At each time *t*, let $N_{z_{1},z_{2}}^{(u),tx}\left(t\right)$ be the number of unicast packets sent by node $z_{1}$ to node $z_{2}$ in the last $T_{avg}$ seconds; let $N_{z_{1},z_{2}}^{(u),rx}\left(t\right)$ be the number of these packets that were received by $z_{2}$; define

(8) $${\hat{p}}_{z_{1},z_{2}}^{\left(u\right)}\left(t\right):=\frac{N_{z_{1},z_{2}}^{(u),rx}\left(t\right)}{N_{z_{1},z_{2}}^{(u),tx}\left(t\right)};$$

recall from (3) that ${\hat{p}}_{z_{1},z_{2}}^{\left(LS\right)}\left(t\right)$ represents the ratio of LS packets transmitted by $z_{1}$; and use these definitions to obtain the minimum variance unbiased linear estimator [53]:

(9) $${\hat{p}}_{z_{1},z_{2}}^{\left(ideal\right)}\left(t\right)=\frac{\sigma_{u}^{2}\left(t\right)}{\sigma_{u}^{2}\left(t\right)+\sigma_{LS}^{2}\left(t\right)}{\hat{p}}_{z_{1},z_{2}}^{\left(LS\right)}\left(t\right)+\frac{\sigma_{LS}^{2}\left(t\right)}{\sigma_{u}^{2}\left(t\right)+\sigma_{LS}^{2}\left(t\right)}{\hat{p}}_{z_{1},z_{2}}^{\left(u\right)}\left(t\right),$$

where $\sigma_{LS}^{2}\left(t\right)$ and $\sigma_{u}^{2}\left(t\right)$ are respectively, the variance of the estimators ${\hat{p}}_{z_{1},z_{2}}^{\left(LS\right)}\left(t\right)$ and ${\hat{p}}_{z_{1},z_{2}}^{\left(u\right)}\left(t\right)$ at time *t*. Considering that the unicast and LS packets have the same size, which is a reasonable approximation when sensors operate in the same manner and the size of data packets is small, the probability of successful transmission of a unicast packet ($p_{z_{1},z_{2}}$) is the same as the probability of successful transmission of a LS packet; and since both $N_{z_{1},z_{2}}^{(u),rx}\left(t\right)$ and $N_{z_{1},z_{2}}^{(LS),rx}\left(t\right)$ are binomial random variables, $\sigma_{u}^{2}\left(t\right)=p_{z_{1},z_{2}}\left[1-p_{z_{1},z_{2}}\right]/N_{z_{1},z_{2}}^{(u),tx}\left(t\right)$ and $\sigma_{LS}^{2}\left(t\right)=p_{z_{1},z_{2}}\left[1-p_{z_{1},z_{2}}\right]/N_{z_{1}}^{(LS),tx}\left(t\right)$. Using these expressions in (9) and simplifying, we obtain

(10) $${\hat{p}}_{z_{1},z_{2}}^{\left(ideal\right)}\left(t\right)=\frac{N_{z_{1},z_{2}}^{(u),rx}\left(t\right)+N_{z_{1},z_{2}}^{(LS),rx}\left(t\right)}{N_{z_{1},z_{2}}^{(u),tx}\left(t\right)+N_{z_{1}}^{(LS),tx}\left(t\right)}.$$

We call the estimator of (10) ideal because the information needed to compute ${\hat{p}}_{ideal}\left(z_{1},z_{2}\right)$ is spread between $z_{1}$ and $z_{2}$: $N_{z_{1},z_{2}}^{(u),tx}\left(t\right)$ is known by $z_{1}$ but not $z_{2}$; and $N_{z_{1},z_{2}}^{(u),rx}\left(t\right)$ and $N_{z_{1},z_{2}}^{(LS),rx}\left(t\right)$ are known by $z_{2}$ but not $z_{1}$. Node $z_{2}$ provides feedback to $z_{1}$: it sends a MAC ACK to every unicast packet sent by node $z_{1}$; and $z_{2}$ broadcasts ${\hat{c}}_{z_{1},z_{2}}^{\left(LS\right)}\left(t\right)$ whenever it sends its LS packet. This feedback is, however, imperfect. Regarding MAC ACK packets sent by $z_{2}$, $z_{1}$ could consider $N_{z_{1},z_{2}}^{(u),rx}\left(t\right)$ as the number of MAC ACK packets received; however, the unicast transmission may have been received by $z_{2}$ with the MAC ACK being lost at $z_{1}$; and $z_{1}$ would consider that the unicast transmission was lost, underestimating $N_{z_{1},z_{2}}^{(u),rx}\left(t\right)$. Regarding $z_{2}$’s LS packet, it may also be lost at $z_{1}$; however, more importantly, $z_{2}$’s LS packet only provides indirect and incomplete information about $N_{z_{1},z_{2}}^{(LS),rx}\left(t\right)$. As explained in Section 3.2, $z_{2}$ computes ${\hat{p}}_{z_{1},z_{2}}^{\left(LS\right)}\left(t\right)$ and uses Equation (2) to compute the 3-bit cost estimate ${\hat{c}}_{z_{1},z_{2}}^{\left(LS\right)}\left(t\right)$. From the quantized ${\hat{c}}_{z_{1},z_{2}}^{\left(LS\right)}\left(t\right)$, node $z_{1}$ is only able to recover a range for ${\hat{p}}_{z_{1},z_{2}}^{\left(LS\right)}\left(t\right)$.

Given the difficulty of applying the ideal estimator, we propose the following practical estimator (The reason for referring to our procedure with the superscript URR will become clear shortly.): let $N_{z_{1},z_{2}}^{(u),ack}\left(t\right)$ be the number of times that $z_{1}$ receives the MAC ACK packet from $z_{2}$ in the last $T_{avg}$ seconds; and $z_{1}$ estimates $p_{z_{1},z_{2}}$ with

(11) $${\hat{p}}_{z_{1},z_{2}}^{\left(URR\right)}\left(t\right)=\frac{N_{z_{1},z_{2}}^{(u),ack}\left(t\right)+{\bar{p}}_{z_{1},z_{2}}^{\left(LS\right)}\left(t\right)\cdot N_{z_{1}}^{(LS),tx}\left(t\right)}{N_{z_{1},z_{2}}^{(u),tx}\left(t\right)+N_{z_{1}}^{(LS),tx}\left(t\right)},$$

where ${\bar{p}}_{z_{1},z_{2}}^{\left(LS\right)}\left(t\right)$ is the highest probability of successful transmissions that maps into the ${\hat{c}}_{z_{1},z_{2}}^{\left(LS\right)}\left(t\right)$ received from $z_{2}$. Table 2 lists ${\bar{p}}_{z_{1},z_{2}}^{\left(LS\right)}\left(t\right)$ from each of the seven possible costs. The values listed were obtained by using Equation (2).

From ${\hat{p}}_{z_{1},z_{2}}^{\left(URR\right)}\left(t\right)$, node $z_{1}$ uses Equation (2) to generate the estimate ${\hat{c}}_{z_{1},z_{2}}^{\left(URR\right)}\left(t\right)$.

Although imperfect, the estimator ${\hat{p}}_{z_{1},z_{2}}^{\left(URR\right)}\left(t\right)$ is able to combine the information from both LS and unicast transmissions and adjust the importance of unicast transmissions as they increase. In other words, before $z_{1}$ sends any unicast transmission to node $z_{2}$, ${\hat{p}}_{z_{1},z_{2}}^{\left(URR\right)}\left(t\right)={\bar{p}}_{z_{1},z_{2}}^{\left(LS\right)}\left(t\right)$, which maps in the same link cost as if we were using the LS-based procedure. When $N_{z_{1},z_{2}}^{(u),tx}\left(t\right)\gg N_{z_{1}}^{(LS),tx}\left(t\right)$, ${\hat{p}}_{z_{1},z_{2}}^{\left(URR\right)}\left(t\right)\approx N_{z_{1},z_{2}}^{(u),ack}\left(t\right)/N_{z_{1},z_{2}}^{(u),tx}\left(t\right)$ and the influence of ${\bar{p}}_{z_{1},z_{2}}^{\left(LS\right)}\left(t\right)$ diminishes. And as $N_{z_{1},z_{2}}^{(u),tx}\left(t\right)\to \infty$, the variance of ${\hat{p}}_{z_{1},z_{2}}^{\left(URR\right)}\left(t\right)\to 0$.

It should be mentioned, however, that ${\hat{p}}_{z_{1},z_{2}}^{\left(URR\right)}\left(t\right)$ is a biased estimate because it estimates a probability different than $p_{z_{1},z_{2}}$. Because ${\hat{p}}_{z_{1},z_{2}}^{\left(URR\right)}\left(t\right)$ uses $N_{z_{1},z_{2}}^{(u),ack}\left(t\right)$, as $N_{z_{1},z_{2}}^{(u),tx}\left(t\right)$ grows, ${\hat{p}}_{z_{1},z_{2}}^{\left(URR\right)}\left(t\right)$ converges to $p_{z_{1},z_{2}}\cdot p_{z_{1},z_{2}}^{\left(ack\right)}$, where $p_{z_{1},z_{2}}^{\left(ack\right)}$ is the probability of successful transmission of the MAC ACK from node $z_{2}$ to node $z_{1}$. This means that route costs and decisions will be taken not based on estimates of $p_{z_{1},z_{2}}$, but instead on estimates of $p_{z_{1},z_{2}}\cdot p_{z_{1},z_{2}}^{\left(ack\right)}$. This is, however, not a problem, because the MAC ACK is needed to complete a transmission in the IEEE 802.15.4 MAC and if $p_{z_{1},z_{2}}^{\left(ack\right)}\ll p_{z_{1},z_{2}}$, then sending packets through node $z_{2}$ would not be a good choice.

Note further that ${\bar{p}}_{z_{1},z_{2}}^{\left(LS\right)}\left(t\right)\cdot N_{z_{1}}^{(LS),tx}\left(t\right)$ is a biased estimate for $N_{z_{1},z_{2}}^{(LS),rx}\left(t\right)$; in fact, it gives an upper bound for $N_{z_{1},z_{2}}^{(LS),rx}\left(t\right)$. This follows because of the 3-bit quantization of cost estimates, which means that there is a range of probabilities that map into the same cost ${\hat{c}}_{z_{1},z_{2}}^{\left(LS\right)}\left(t\right)$ received from node $z_{2}$; therefore, there is a range of possible $N_{z_{1},z_{2}}^{(LS),rx}\left(t\right)$ for a given ${\hat{c}}_{z_{1},z_{2}}^{\left(LS\right)}\left(t\right)$ received at node $z_{1}$. The lower bound for $N_{z_{1},z_{2}}^{(LS),rx}\left(t\right)$ reduces ${\hat{p}}_{z_{1},z_{2}}^{\left(URR\right)}\left(t\right)$, and thus, increase ${\hat{c}}_{z_{1},z_{2}}^{\left(URR\right)}\left(t\right)$; likewise, using the upper bound for $N_{z_{1},z_{2}}^{(LS),rx}\left(t\right)$ decreases ${\hat{c}}_{z_{1},z_{2}}^{\left(URR\right)}\left(t\right)$. Thus, using any value lower than $N_{z_{1},z_{2}}^{(LS),rx}\left(t\right)$ may cause ${\hat{c}}_{z_{1},z_{2}}^{\left(URR\right)}\left(t\right)$ to be higher than it would be if $N_{z_{1},z_{2}}^{(LS),rx}\left(t\right)$ are known at node $z_{1}$. As will become clear in the next section, if a route has a chance of being the best route, we would like to select it to send a batch of unicast packets on the route to improve our cost estimate ${\hat{c}}_{z_{1},z_{2}}^{\left(URR\right)}\left(t\right)$. Thus, using the upper bound for $N_{z_{1},z_{2}}^{(LS),rx}\left(t\right)$ prevents the exclusion of a route that would otherwise be selected if $N_{z_{1},z_{2}}^{(LS),rx}\left(t\right)$ were to be known at node $z_{1}$. It should be noted that, as mentioned in the previous paragraph, the estimate for $N_{z_{1},z_{2}}^{(LS),rx}\left(t\right)$ becomes less and less relevant as $N_{z_{1},z_{2}}^{(u),tx}\left(t\right)$ grows, which means that this estimate is relevant only while $N_{z_{1},z_{2}}^{(u),tx}\left(t\right)$ is small.

Since our proposed estimator ${\hat{p}}_{z_{1},z_{2}}^{\left(URR\right)}\left(t\right)$ depends on the number $N_{z_{1},z_{2}}^{(u),tx}\left(t\right)$ of unicast packets sent from $z_{1}$ to $z_{2}$, $z_{1}$ needs to first select node $z_{2}$ as the next hop towards the concentrator in order to start sending unicast packets to it. Furthermore, when node $z_{1}$ has to select among multiple routes to the concentrator, it needs good estimates for the probability of successful transmission to each of the next hop candidates in order to build the various route costs. In other words, if node $z_{1}$ has nodes $z_{2}$ and $z_{3}$ as potential candidates to reach the concentrator, $z_{1}$ would have to send some packets through $z_{2}$ and other packets through $z_{3}$ in order to be able to obtain good estimates ${\hat{p}}_{z_{1},z_{2}}^{\left(URR\right)}\left(t\right)$ and ${\hat{p}}_{z_{1},z_{3}}^{\left(URR\right)}\left(t\right)$. For this, we propose the route selection procedure discussed next.

### 6.2. Modified Route Selection Procedure

Recall from Section 2.2 that ZigBee specifies that nodes compare the costs of candidate routes and select the next hop towards the concentrator whenever they receive a RREQ packet. In order to remain compliant with the ZigBee protocol, our modified route selection procedure still selects routes whenever RREQ packets are received; however, our route selection procedure has an additional treatment to decide among routes with the same cost.

**Figure 3:**
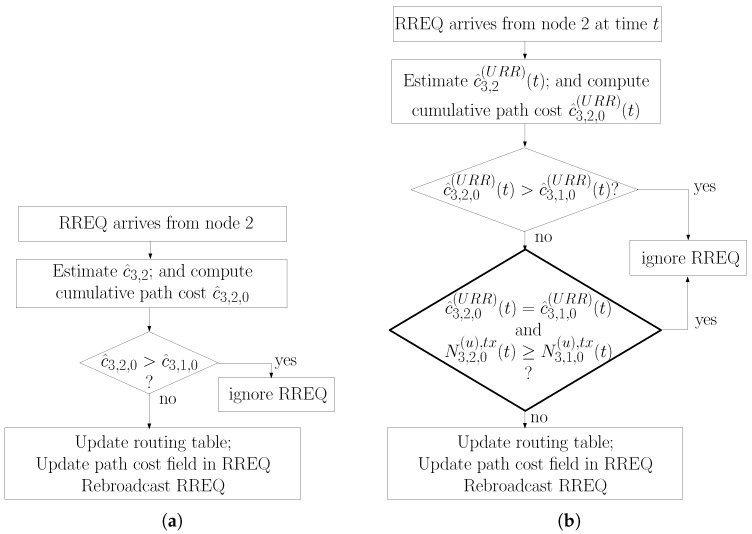
(**a**) ZigBee’s treatment of a route request (RREQ) message in the M2O routing procedure; and (**b**) modified treatment of a RREQ message in the U-RR procedure. This figure illustrates the treatment of a RREQ arriving from node 2 at node 3. Node 3 has previously received a RREQ from node 1 and stored node 1 and the estimated cumulative route cost of the route 3,1,0 in its routing table.

The original ZigBee route selection procedure and our proposed route selection procedure are illustrated in Figure 3a,b respectively. Comparing these figures, it is possible to notice that our procedure starts processing an incoming RREQ in the same way as in the ZigBee specification: whenever a node $z_{1}$ receives an incoming RREQ from a node $z_{2}$, it computes the cumulative route cost toward the concentrator. If such a cost is lower than the cumulative route cost of the current next hop node, then node $z_{1}$ updates its routing table to reflect node $z_{2}$ as the next hop toward the concentrator. If such a cost is greater than the cumulative route cost of the current next hop node, then node $z_{1}$ disregards the RREQ.

The novelty of the proposed procedure is in the treatment when two or more next hop candidates have the same cumulative route cost, in the second test of Figure 3b. To understand this step and its effect, consider the scenario of Figure 1; assume that node 3 has node 1 as the current next hop towards the concentrator and assume node 3 receives a RREQ from node 2 with the same cumulative route cost as node 1. Recall that $N_{3,1}^{(u),tx}\left(t\right)$ and $N_{3,2}^{(u),tx}\left(t\right)$ refer to the number of unicast packets sent by node 3 on the last $T_{avg}$ seconds to nodes 1 and 2 respectively. If $N_{3,2}^{(u),tx}\left(t\right)\geq N_{3,1}^{(u),tx}\left(t\right)$, then node 3 disregards the RREQ and node 1 remains as the next hop toward the concentrator. However, if $N_{3,2}^{(u),tx}\left(t\right)<N_{3,1}^{(u),tx}\left(t\right)$, then node 3 selects node 2 as the next hop. The reason for this is to enable node 3 to collect more samples from the link 3,2 in order to obtain a low variance estimate for ${\hat{p}}_{3,2}^{\left(URR\right)}\left(t\right)$ in subsequent RREQ cycles.

As subsequent RREQ packets with the same cumulative route cost from other next hop candidates arrive, the procedure performs the same comparison. In effect, among the next hop candidates that have the same cumulative route cost, node 3 selects the next hop candidate with lowest number of unicast packets transmitted during the last $T_{avg}$ seconds.

A second non-trivial aspect of the second test of Figure 3b is that it causes a node to, in effect, select multiple routes to the concentrator over multiple RREQ intervals. To understand this aspect, assume in the illustration of the previous paragraph that $N_{3,2}^{(u),tx}\left(t\right)<N_{3,1}^{(u),tx}\left(t\right)$, causing node 3 to select node 2 as the next hop. This causes node 3 to stop sending the application packets to the concentrator using node 1 as next hop and start sending them using node 2. This causes $N_{3,2}^{(u),tx}\left(t\right)$ to increase, and as previous transmissions using node 1 fall out of the averaging window $T_{avg}$, causes $N_{3,1}^{(u),tx}\left(t\right)$ to decrease. Eventually, in subsequent RREQ intervals, $N_{3,2}^{(u),tx}\left(t\right)>N_{3,1}^{(u),tx}\left(t\right)$ and node 3 selects node 1 as next hop towards the concentrator. This results in node 3 selecting each next hop candidate with the same cumulative route cost in a round-robin fashion. This aspect is beneficial in that it increases the redundancy of the system, avoids that a certain set of nodes be overused, and to the point of this paper, allows a node to proper measure the link costs of multiple nodes.

Because our procedure uses unicast packets to improve the link cost estimation and selects routes in round-robin fashion, we shall refer to it as the **U-RR procedure**.

With respect to existing models, the use of unicast transmissions in our U-RR procedure is similar to [22,24,25]; however, our U-RR procedure is tailored to ZigBee and its 3-bit link cost quantization.

It is important to highlight that our U-RR procedure works as the ZigBee specified procedure when the cumulative route cost offered by the RREQ sender is different than the cost offered by the current next hop node. As such, the U-RR procedure is able to maintain the qualities of the existing ZigBee procedure, improving it only when there is a tie between next hop candidates, which is fairly common in ZigBee because of its 3-bit link cost quantization.

An additional important point is that, in the existing ZigBee procedure, the choice between next hop candidates with the same cumulative cost is random: a node would choose as next hop, the node whose RREQ arrived first. In the U-RR procedure, the choice between next hop candidates with the same cumulative cost is no longer random, being guided by the number of past packets sent to each candidate.

### 6.3. Implementation Considerations

To implement the U-RR procedure, the following must be implemented in ZigBee nodes:

- Nodes need to track the number of packets transmitted and the number of packets acknowledged with each neighbor node separately. This information should be stored within the network layer to enable access by the route selection function. The network layer already has provisions for a neighbor list [13], which could be expanded to store this additional information. Ideally, the time of each transmission would be stored in order to determine when transmission records become older than the averaging window. Results that follow assume this ability. Alternatively, the tracking of the number of packets successfully transmitted could be implemented with a cyclic buffer, where the result of the latest transmission would overwrite the result of the oldest transmission in the buffer.
- The IEEE 802.15.4 MAC layer must be augmented so that it provides the network layer with the number of retransmissions needed to transmit a packet. This would probably be done in the service application point and the MAC would provide this information in additional fields of the MCPS-Data.Confirm message. Although provisioning of such information is not forecasted by the IEEE 802.15.4 MAC specification, it is possible for manufacturers to offer additional information in their service access points while still complying with the IEEE 802.15.4 specification.
- The treatment of the RREQ at the network layer would have to be augmented to follow the procedure of Figure 3b.

It is important to mention that the U-RR procedure does not require any changes to the ZigBee protocol; i.e., there is no need for new protocol messages nor changes to protocol frame formats.

## 7. Performance Evaluation

### 7.1. Symmetric Topologies

We first evaluated our procedure in symmetric scenarios in order to evaluate whether it can reduce the variance observed in the LS-based procedure.

#### 7.1.1. Scenario S1: ZigBee-Only, One-Hop Routes

Consider first the topology of Figure 1 in which sensors at node 3 at (0,−80), node 4 at (−130,0), and node 5 at (130,0) send sensor measurements to the concentrator 0 at (0,80); and node 1 at (−35,0) and node 2 at (35,0) are available for routing packets. This is the same scenario as considered in the examples of Section 5.1 and it is reproduced in Figure 1 to facilitate the understanding.

For this scenario, assume that nodes 3, 4, and 5 generate the same average number of packets/s. Because of the symmetry of the scenario, the actual route costs for routes 3,1,0 and 3,2,0 are the same. To see why $c_{3,1,0}=c_{3,2,0}$, recall that packet losses occur due to low SINR. Using the ns3 channel model, the distances between nodes are such that the probability of a successful transmission is very close to 1 when no interferers transmit, meaning that packet losses are occurring mainly due to interfering transmissions. Interfering transmissions occur randomly due to the various protocol procedures, such as MAC random backoff or random delays in application packet generation. In this symmetric scenario, the rates of interfering transmissions in nodes 1 and 2 are the same, causing $p_{3,1}=p_{3,2}$. Likewise, the rates of interfering transmissions in node 0 are the same if either node 1 or node 2 transmits, causing $p_{1,0}=p_{2,0}$; and $c_{3,1,0}=c_{3,2,0}$.

We first evaluate the performance of the U-RR procedure in the single simulation run of the Example 1 of Section 5.1, in which nodes 3, 4, and 5 generate an average of 20 packets/s; Figure 2e in Section 5.1 illustrates the cumulative costs ${\hat{c}}_{3,1}^{\left(URR\right)}\left(t\right)$ and ${\hat{c}}_{3,2}^{\left(URR\right)}\left(t\right)$ measured at node 3 when using the U-RR procedure. Comparing this figure with Figure 2a, which refers to the same scenario but using the LS-based procedure, it is possible to observe that the U-RR procedure was able to generate estimates with less variation and better recognize that routes 3,1,0 and 3,2,0 have the same cost.

To better analyze the performance of the U-RR procedure in reducing the variance of cost estimates, we varied the traffic level generated by nodes 3, 4, and 5, and at each traffic level, we repeated the simulation 30 times, each time with a different random seed.

For each simulation run and each estimation procedure, we simulated the network for 400 s and collected the estimated route costs ${\hat{c}}_{3,1,0}\left(t\right)$ and ${\hat{c}}_{3,2,0}\left(t\right)$ for t≥81 s. Let $N_{rreq}^{rx}$ be the number of RREQ messages received at node 3 in the time interval [81,400]; and let ${\left\{t_{n}\right\}}_{n=1}^{N_{rreq}^{rx}}$ be the times in which the RREQ were received. From these estimated route costs, we computed the following performance metrics:

- The average estimated cost measured at node 3 for the routes 3,1,0 and 3,2,0. For example, for the route 3,2,0:
  (12) $$\begin{array}{ccc} {\bar{c}}_{3,2,0} & := & \frac{1}{N_{rreq}^{rx}}\sum_{n=1}^{N_{rreq}^{rx}}{\hat{c}}_{3,2,0}\left(t_{n}\right). \end{array}$$
- The standard deviation of the estimated costs measured at node 3 for the routes 3,1,0 and 3,2,0. For example, for the route 3,2,0:
  (13) $$\begin{array}{ccc} \sigma_{3,2,0} & := & \sqrt{\frac{1}{N_{rreq}^{rx}-1}\sum_{n=1}^{N_{rreq}^{rx}}{\left({\hat{c}}_{3,2,0}\left(t_{n}\right)-{\bar{c}}_{3,2,0}\right)}^{2}}.. \end{array}$$
  The measures $\sigma_{3,1,0}$ and $\sigma_{3,2,0}$ tell us how much the cost of each route varied over the course of the simulation run.

**Figure 4:**
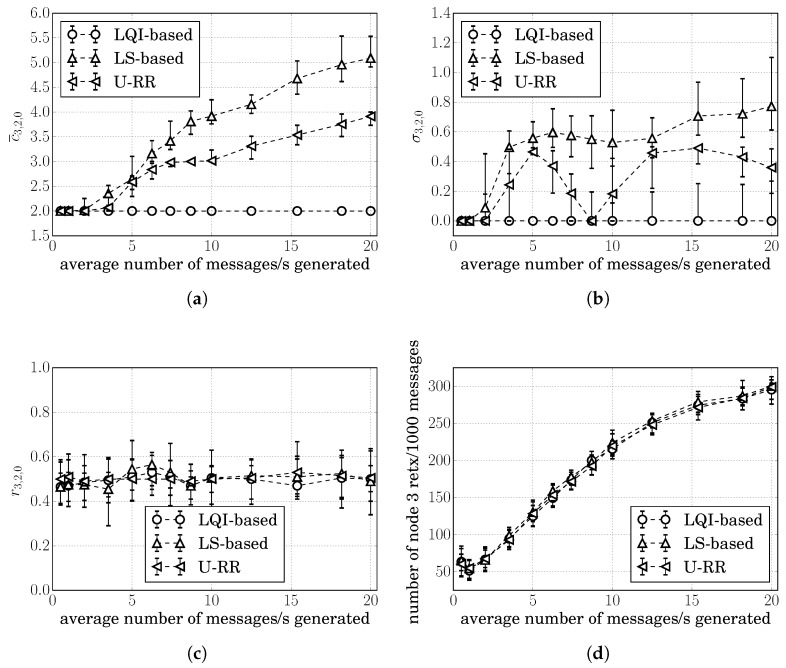
The median and the 15th to 85th percentiles of (**a**): the cumulative route cost of route 3,2,0 (${\bar{c}}_{3,2,0}$), (**b**): the standard deviation of the estimated costs measured at node 3 for the route 3,2,0 ($\sigma_{3,2,0}$), (**c**): the ratio of times that route 3,2,0 was chosen ($r_{3,2,0}$), and (**d**): the number of times that node 3 had to retransmit for every 1000 messages for the symmetric scenario S1.

Figure 4a,b respectively, show the median of ${\bar{c}}_{3,2,0}$ and $\sigma_{3,2,0}$ for 30 simulation runs at each traffic level for the LS-based, the LQI-based, and the U-RR procedures. Error bars represent the range between the 15 and 85 percentiles among 30 simulation runs. The figures for the median of ${\bar{c}}_{3,1,0}$ and $\sigma_{3,1,0}$ showed similar behavior and were therefore omitted.

As discussed in Section 5, the LQI-based procedure produced the lowest variation in all, with the median of $\sigma_{3,2,0}^{\left(LQI\right)}\approx 0$; however, it was insensitive to the traffic load, as shown in Figure 4a: the median of ${\bar{c}}_{3,2,0}^{\left(LQI\right)}\approx 2$ for all traffic loads.

From Figure 4b, it is possible to see that, while all procedures showed $\sigma_{3,2,0}\approx 0$ at low traffic loads, the U-RR procedure consistently produced cost estimates with less variation than the LS-based procedure as the traffic load increased above 2 packets/s.

The better performance of the U-RR procedure in comparison with the LS-based procedure is justified by the U-RR use of both LS and unicast packets to estimate the probability of successful transmission in links. When the traffic load was low, the U-RR procedure relied mostly on the LS transmissions to estimate the link cost, as can be observed in (11). When the traffic load increased, the number of unicast transmissions became much higher than the number of LS transmissions and the U-RR procedure used these additional transmissions to reduce the variation of cost estimates.

It is interesting to observe in Figure 4b that the median of $\sigma_{3,2,0}^{\left(URR\right)}$ increased after 2 packets/s, reached a peak at 5 packets/s, reduced to 0 at 9 packets/s, and again increased after 9 packets/s. To understand this behavior, recall that estimated costs are rounded to the nearest integer. If the true cost is between two integers, the estimated cost would be alternating between the two integers, increasing the standard deviation of cost estimates. This rounding effect can be seen in Figure 4a,b: at 5 packets/s, the median ${\bar{c}}_{3,2,0}^{\left(URR\right)}\approx 2.5$ and the median $\sigma_{3,2,0}^{\left(URR\right)}$ increased; at 9 packets/s, the median ${\bar{c}}_{3,2,0}^{\left(URR\right)}\approx 3$ and the median $\sigma_{3,2,0}^{\left(URR\right)}$ was close to 0.

Figure 4c shows how often route 3,2,0 was chosen by each procedure, illustrating that all procedures chose route 3,2,0 around 50% of the times. This behavior was expected since $c_{3,1,0}=c_{3,2,0}$.

Since route decisions can impact the probability of retransmissions, we also computed the number of times that node 3 had to retransmit a packet every 1000 messages generated above the network layer. Since $c_{3,1,0}=c_{3,2,0}$ in this scenario, all of the procedures showed similar results, as illustrated in Figure 4d.

#### 7.1.2. Scenario S2: WiFi interference

**Figure 5:**
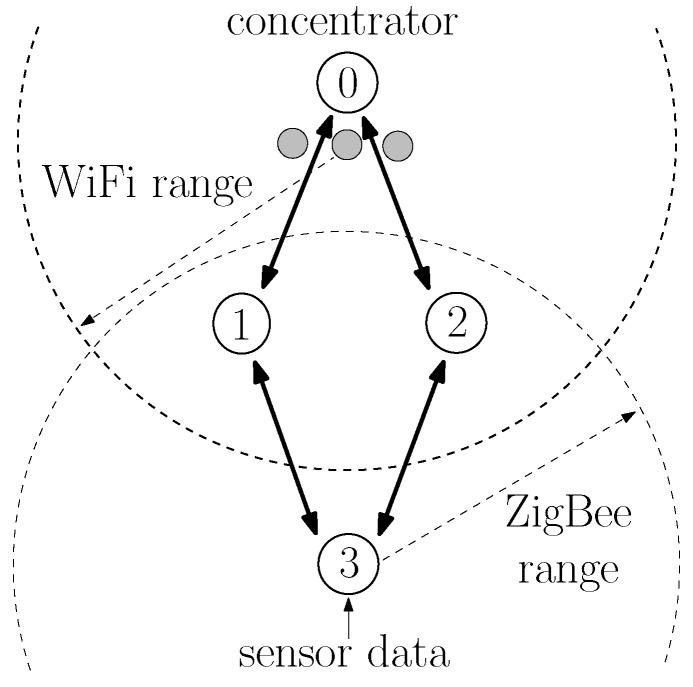
Topology where ZigBee devices share same spectrum as WiFi devices.

Consider a symmetric scenario with WiFi interference. As illustrated in Figure 5, ZigBee nodes 0, 1, 2, and 3 are respectively at (0,80), (−35,0), (35,0), and (0,−80) and node 3 sends application data at an average rate of 20 packets/s to node 0 in the same communication channel as a WiFi IEEE 802.11n network. The WiFi network consists of one access point and two stations respectively, at (0,60), (10,60), and (−10,60). We assume that, at the time 100 s of the simulation, the WiFi access point starts two data streams of constant-bit-rate traffic of $R_{WiFi}$ bits/s, one to each WiFi station; and these streams last until the end of the simulation at 400 s.

In this topology, the WiFi devices are far from node 3 and may transmit while node 3 is transmitting, causing interference in the reception at nodes 1 and 2. As explained in Section 7.1.1, the symmetry of the topology means that the rates of interfering transmissions in nodes 1 and 2 are the same and the actual route costs for routes 3,1,0 and 3,2,0 satisfy $c_{3,1,0}=c_{3,2,0}$.

**Figure 6:**
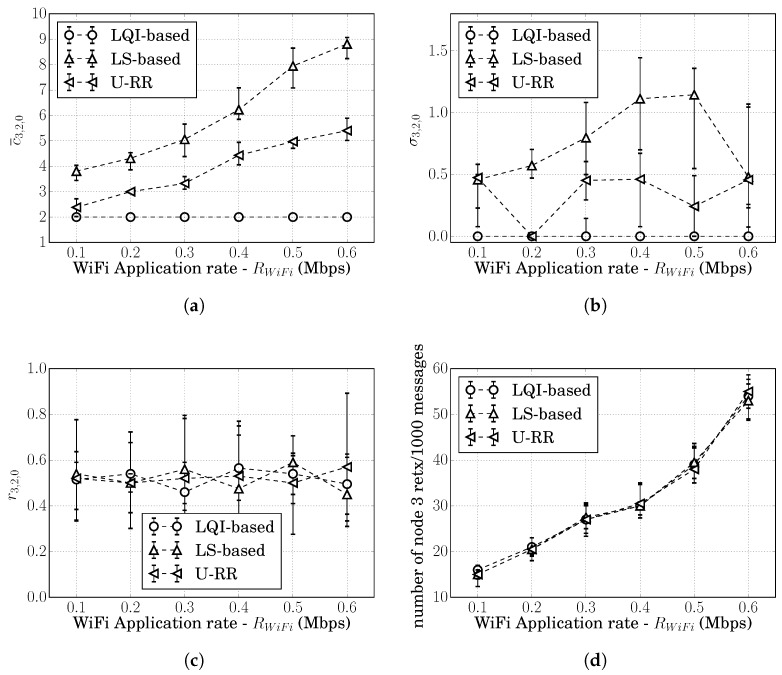
The median and the 15th to 85th percentiles of (**a**): the cumulative route cost of route 3,2,0 (${\bar{c}}_{3,2,0}$), (**b**): the standard deviation of the estimated costs measured at node 3 for the route 3,2,0 ($\sigma_{3,2,0}$), (**c**): the ratio of times that route 3,2,0 was chosen ($r_{3,2,0}$), and (**d**): the number of times that node 3 had to retransmit for every 1000 messages for the symmetric scenario S2.

Figure 6a,b respectively, show the median and the 15th to 85th percentiles of ${\bar{c}}_{3,2,0}$ and $\sigma_{3,2,0}$ for 30 simulation runs at each WiFi application rate ($R_{WiFi}$) for the LS-based, the LQI-based, and the U-RR procedures. Since the WiFi traffic started only at t=100 s, the ${\bar{c}}_{3,2,0}$ and $\sigma_{3,2,0}$ were obtained considering cost estimates after t=181 s. The figures for the median of ${\bar{c}}_{3,1,0}$ and $\sigma_{3,1,0}$ showed similar behavior, and were therefore, omitted.

As discussed previously, the LQI-based procedure had the lowest variation in all scenarios; but it was not able to detect the WiFi interference. Even when the WiFi traffic was consuming more than 20% of the channel time, ${\bar{c}}_{3,1,0}^{\left(LQI\right)}={\bar{c}}_{3,2,0}^{\left(LQI\right)}=2$ most of the time.

Comparing the LS-based and the U-RR procedures, similarly to the analysis in the ZigBee-only network, it is possible to observe in Figure 6b that $\sigma_{3,2,0}^{\left(URR\right)}\leq \sigma_{3,2,0}^{\left(LS\right)}$ in most traffic loads. The better performance of the U-RR procedure in comparison to the LS-based procedure was due to its reliance on unicast transmissions, which were more abundant than LS transmissions. It is also possible to observe the cyclic variations in $\sigma_{3,2,0}^{\left(URR\right)}$ caused by the rounding of cost estimates discussed in the previous section. These results show that the U-RR procedure is able to reduce cost estimate variations even under WiFi interference.

It is interesting to note in Figure 6b that $\sigma_{3,2,0}^{\left(LS\right)}\approx \sigma_{3,2,0}^{\left(URR\right)}$ when $R_{WiFi}=600$ kbps. To understand this, we observed that the interference in the links between nodes 0, 1, and 2 caused by the WiFi transmissions when $R_{WiFi}=600$ kbps caused both ${\hat{p}}_{1,0}^{\left(LS\right)}\left(t\right)$ and ${\hat{p}}_{2,0}^{\left(LS\right)}\left(t\right)$ to be below 0.627, which maps into the maximum link cost 7. This can be seen in Figure 6a, which shows ${\hat{c}}_{3,2,0}^{\left(LS\right)}\left(t\right)\approx 8.7$ when $R_{WiFi}=600$ kbps. This suggests that the high WiFi traffic caused the link cost estimates ${\hat{c}}_{1,0}^{\left(LS\right)}\left(t\right)$ and ${\hat{c}}_{2,0}^{\left(LS\right)}\left(t\right)$ to saturate at 7, reducing the standard deviation of the cost estimates over the course of the simulation.

Also similar to the Scenario S1, all procedures choose the route 3,2,0 around 50% of the time and showed similar performance regarding retransmissions, as illustrated in Figure 6c,d. This behavior was expected, since $c_{3,1,0}=c_{3,2,0}$.

#### 7.1.3. Scenario S3: Two-Hop Scenario

**Figure 7:**
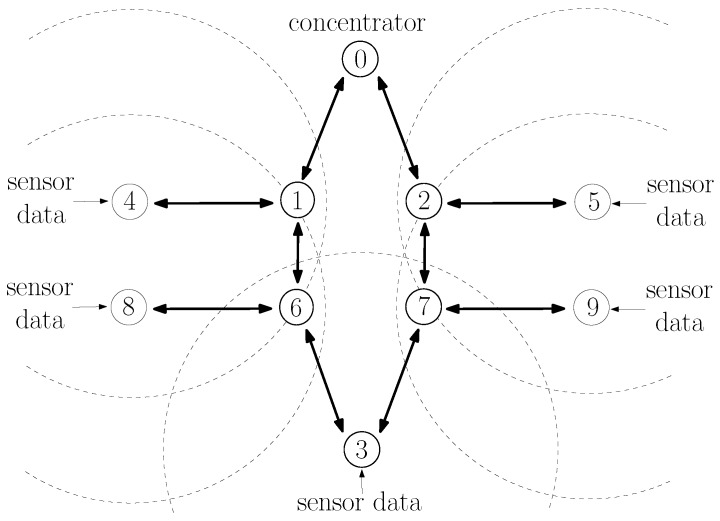
Topology with one-hop and two-hop routes.

Consider now the topology of Figure 7 involving routes with more than 1 hop. In this scenario, sensors at node 3 at (0,−140), node 4 at (−130,0), node 5 at (130,0), node 8 at (−130,−60), and node 9 at (130,60) send sensor measurements to the concentrator 0 at (0,80); and node 1 at (−35,0), node 2 at (35,0), node 6 at (−35,60), and node 7 at (35,−60) are available for routing packets. Because of their distance, nodes 4 and 8 are hidden from nodes 5 and 9 and vice-versa; and nodes 4, 5, 8, and 9 are hidden from nodes 0 and 3 and vice-versa. Node 1 is not close enough to be within node 8’s communication range; however, it can detect transmissions from node 8. Likewise, node 2 can detect transmissions from node 9; node 6 can detect transmissions from node 4; and node 7 can detect transmissions from node 5. Consider also the same parameters as described in Section 5. For this scenario, assume that nodes 3, 4, 5, 8, and 9 generate the same average number of packets/s.

Because of the symmetry of this scenario, the rates of interfering transmissions in nodes 1, 2, 6, and 7 are the same; and, as explained in Section 7.1.1, $c_{3,6,1,0}=c_{3,7,2,0}$ and $c_{3,6,2,0}=c_{3,7,1,0}$; however, $c_{3,7,2,0}<c_{3,7,1,0}$. To see this, note first that node 7 is farther from node 1 than it is from node 2. Furthermore, and perhaps more importantly, node 7 is hidden from node 4’s transmissions, while it is not hidden from node 5’s transmissions, which means a lower probability of successfully transmitting to node 1; i.e., $p_{7,1}<p_{7,2}$. Same argument follows to justify $c_{3,6,1,0}<c_{3,6,2,0}$.

Since $c_{3,6,1,0}=c_{3,7,2,0}<c_{3,6,2,0}=c_{3,7,1,0}$, it is desirable to choose either routes 3,6,1,0 or 3,7,2,0. Note that the choice of a route is not done by node 3 alone. As explained in Section 2.2, a node does not select the whole route toward the concentrator; instead, it only selects the next hop node; and the next hop node then chooses its next hop node toward the concentrator. All nodes operate the same next hop selection procedure and we will refer to a procedure as *selecting* a route as the route that resulted by the distributed operation of the procedure in the various nodes of the network.

**Figure 8:**
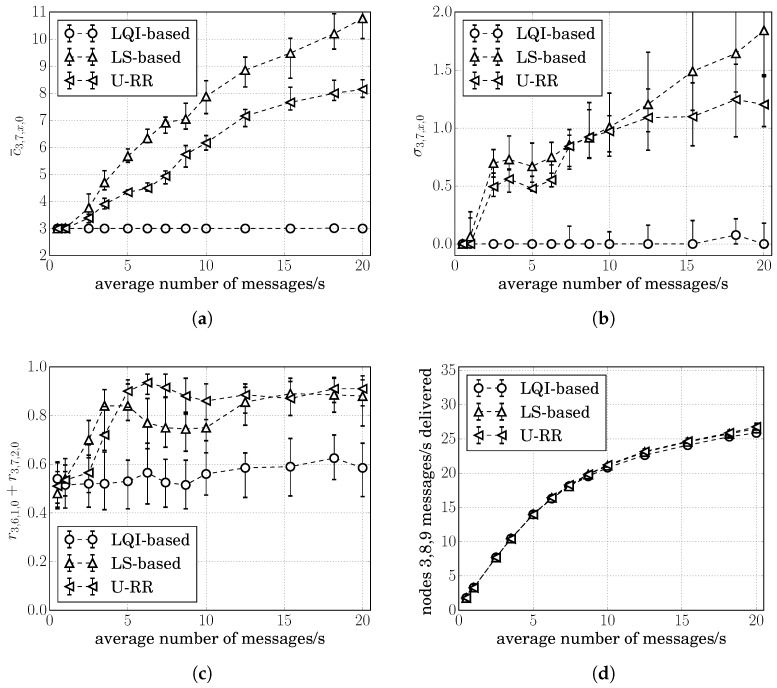
The median and the 15th to 85th percentiles of (**a**): the cumulative route cost of routes 3,7,x,0 (${\bar{c}}_{3,7,x,0}$), (**b**): the standard deviation of the estimated costs measured at node 3 for the routes 3,7,x,0 ($\sigma_{3,7,x,0}$), (**c**): the ratio of times that packets were relayed by the best routes 3,6,1,0 and 3,7,2,0 ($r_{3,6,1,0}+r_{3,7,2,0}$), and (**d**): the delivery rate of messages originated at nodes 3, 8, and 9 in the symmetric scenario S3.

Figure 8a,b show the average of cumulative cost estimates ${\bar{c}}_{3,7,x,0}$ and $\sigma_{3,7,x,0}$. We use ’*x*’ in ${\bar{c}}_{3,7,x,0}$ and $\sigma_{3,7,x,0}$ because node 3 cannot differentiate a RREQ arriving through the route 3,6,1,0 from a RREQ arriving through the route 3,6,2,0; therefore, node 3 cannot estimate $c_{3,7,1,0}$ or $c_{3,7,2,0}$ separately. The figures showing ${\bar{c}}_{3,6,x,0}$ and $\sigma_{3,6,x,0}$ followed similar behavior as shown in Figure 8a,b, and were therefore, omitted.

It is possible to see in Figure 8a,b that many of the conclusions reached for the single-hop scenario were also present in this two-hop scenario: the LQI-based procedure had the lowest variation in its cost estimates; however, it was not able to detect traffic increases; all procedures behaved similarly when the traffic load was low; and $\sigma_{3,7,x,0}^{\left(URR\right)}\leq \sigma_{3,7,x,0}^{\left(LS\right)}$ as the traffic increased above 2 packets/s.

Letting $r_{3,6,1,0}$ and $r_{3,7,2,0}$ respectively, denote the ratio of times that the resulting route was 3,6,1,0 and 3,7,2,0; the sum $r_{3,6,1,0}+r_{3,7,2,0}$ represents the ratio of time that a procedure chose one of the best routes. Figure 8c shows the average of $r_{3,6,1,0}+r_{3,7,2,0}$ for 30 simulation runs at each traffic level for the LS-based, the LQI-based, and the U-RR procedures.

It is possible to see in Figure 8c that the LS-based procedure resulted in better route decisions than both the U-RR and the LQI-based procedures when the average number of packets/s generated by nodes was between 1 and 5 packets/s. To understand this, recall that the U-RR procedure selects the next hop node with the least number of packets transmitted when two RREQs arrive with the same cumulative cost. When the traffic load is low, packet losses due to the hidden node problem are low, and although $c_{3,6,1,0}<c_{3,6,2,0}$, the sum of 3-bit quantized link costs may result in the same value, and the U-RR procedure would result in node 6 selecting node 2 as the next hop towards node 0 more often to better estimate its actual cost.

However, when the average number of packets/s generated by nodes was between 5 and 12.5 packets/s, the U-RR procedure resulted in better route selections than both the LQI-based and the LS-based procedures. In some cases, the U-RR procedure chose one of the preferred routes 3,6,1,0 and 3,7,2,0 more than 90% of the time, while the LQI-based and LS-based procedures selected the preferred routes around 55% and 75% of the time respectively.

When the average number of packets/s generated by nodes was very large (above 12.5 packets/s in this case), both the LS-based and the U-RR procedure reached similar performances. To understand this, note that higher traffic loads increase the frequency of hidden node problems. If the traffic load is high enough, the difference in cost of candidate routes becomes wide enough that, even with the higher variation of the LS-based procedure, the noisy cost estimate of routes 3,6,1,0 or 3,7,2,0 is still above the noisy cost estimate of routes 3,6,2,0 or 3,7,1,0.

Lastly, since $c_{3,6,1,0}=c_{3,7,2,0}<c_{3,6,2,0}=c_{3,7,1,0}$, we also evaluated the impact of choosing the suboptimal routes 3,6,2,0 or 3,7,1,0 in the packet delivery rate of each procedure. Since the routes were not being chosen directly by node 3, we computed the rate of successfully delivering messages. Also, because the procedures also select between route 8,6,1,0, route 8,6,2,0, route 9,7,1,0, and route 9,7,2,0, we computed the rate of successfully delivering packets from nodes 3, 8, and 9 for each procedure. As illustrated in Figure 8d, it is possible to note that the U-RR procedure performed slightly better than the LQI-based procedure when the traffic load was large; however, the difference with respect to the LS-based procedure was small, suggesting that the amount of asymmetry was not large enough to cause an impact in the network delivery performance.

### 7.2. Asymmetric Topologies

We also evaluated our procedure in asymmetric scenarios in order to evaluate how well it improves the selection of the best route available. Given that the three estimation procedures perform similarly at low traffic loads, we focused on high traffic load conditions.

#### 7.2.1. Scenario A1: ZigBee-Only, One-Hop Routes

Consider again the topology of Figure 1 as in Scenario S1, but now assume that node 4 generates more traffic load than node 5, making the rate of interfering transmissions in node 1 greater than the rate of interfering transmissions in node 2, which results in $c_{3,1,0}>c_{3,2,0}$. In this case, it is desirable that node 3 chooses the route 3,2,0 as much as possible to avoid the hidden node problem caused by node 4.

We first evaluate the performance of the U-RR procedure in the single simulation run of the Example 2 of Section 5.1, in which nodes 3, 4, and generate an average of 20, 10, and 0.5 packets/s respectively. Figure 2f in Section 5.2 illustrates the cumulative costs measured at node 3 when using the U-RR procedure. Comparing this figure with Figure 2b, which refers to the same scenario but using the LS-based procedure, it is possible to observe that the U-RR procedure was able to generate estimates with less variation and better recognize that route 3,2,0 had lower cost than route 3,1,0 given the higher traffic caused by node 4 upon node 1.

To better analyze the performance of the U-RR procedure at selecting the best route, we varied the traffic level generated by node 5, and at each traffic level, we repeated the simulation 30 times, each time with a different random seed.

**Figure 9:**
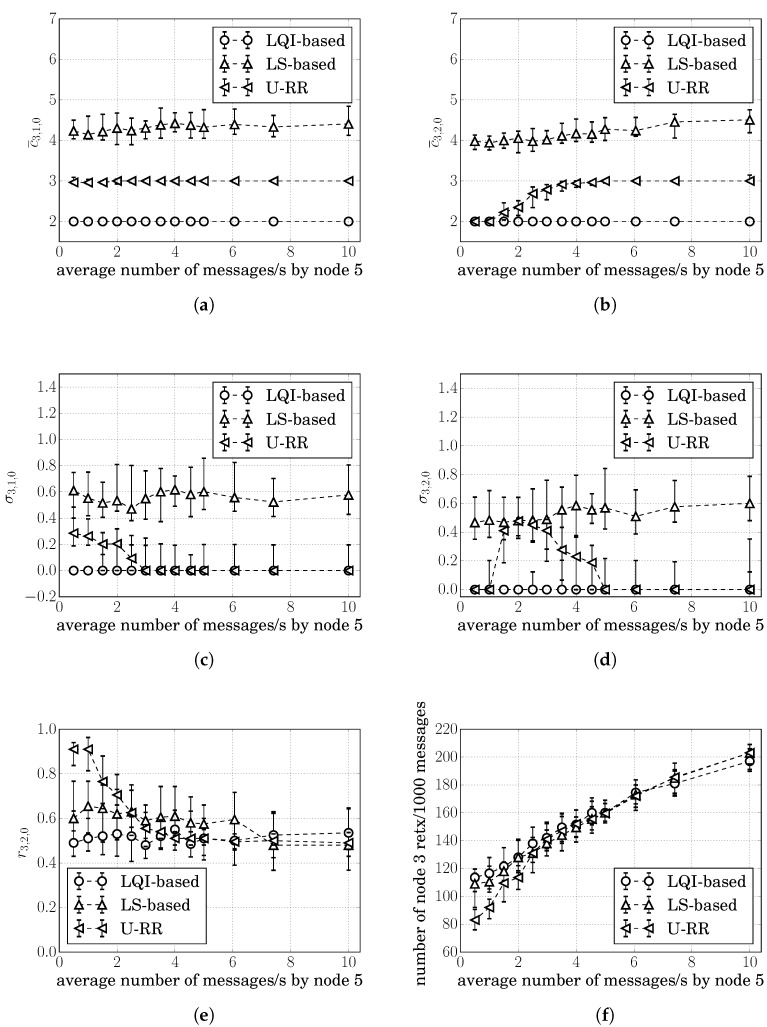
The median and the 15th to 85th percentiles of (**a**): the cumulative route costs of the route 3,1,0 (${\bar{c}}_{3,1,0}$), (**b**): the cumulative route costs of the route 3,2,0 (${\bar{c}}_{3,2,0}$), (**c**): the standard deviation of the estimated costs measured at node 3 for the route 3,1,0 ($\sigma_{3,1,0}$), (**d**): the standard deviation of the estimated costs measured at node 3 for the route 3,2,0 ($\sigma_{3,2,0}$), (**e**): the ratio of times that route 3,2,0 was chosen ($r_{3,2,0}$), and (**f**): the number of times that node 3 had to retransmit per 1000 messages generated in the scenario A1.

Figure 9a–f respectively, show the median and the 15th to 85th percentiles of ${\bar{c}}_{3,1,0}$, ${\bar{c}}_{3,2,0}$, $\sigma_{3,1,0}$, $\sigma_{3,2,0}$, $r_{3,2,0}$, and node 3’s number of retransmissions per 1000 messages generated for 30 simulation runs at each of node 5’s traffic level for the LS-based, the LQI-based, and the U-RR procedures.

Regarding the LQI-based procedure, as shown in Figure 9a,b, it estimated both routes to have cost 2 regardless of the traffic load generated by node 5. This means that node 3 chose the best route 3,2,0 randomly, around 50% of the times, as shown in Figure 9e.

Regarding the LS-based procedure, it struggled to recognize route 3,2,0 as the best route. Although Figure 9a,b shows that the LS-based procedure resulted in ${\bar{c}}_{3,1,0}>{\bar{c}}_{3,2,0}$, the difference was within the standard deviation of the cost estimates, as shown in Figure 9c,d. The higher variation of cost estimates caused the LS-based procedure to select route 3,2,0 only 60% of the time when node 5 generated only 0.5 packets/s, as shown in Figure 9e.

In contrast, as illustrated in Figure 9a through Figure 9e, when node 5 generated 0.5 packets/s, the differences in cost estimates of the U-RR procedure were much higher than the standard deviation, and the U-RR procedure chose route 3,2,0 only 89% of the times. The U-RR procedure also resulted in a lower number of retransmissions: as shown in Figure 9f, the median number of node 3 retransmissions per 1000 messages was 115 when using the LQI-based procedure, 110 when using the LS-based procedure, and 83 when using the U-RR procedure.

As the traffic load generated by node 5 increased, the true cost $c_{3,2,0}$ started to increase above the cost 2; however, because of the cost rounding discussed before, the cost estimates by the U-RR procedure started to alternate between cost 2 and cost 3, which is the cost of route 3,1,0, and $r_{3,2,0}^{\left(URR\right)}$ decreased little by little, until it reached 50% when node 5’s traffic load reached 4 packets/s.

It is important to observe that, when the node 5 generated 4 packets/s, the actual unrounded cost of the route 3,1,0 was still higher than the actual unrounded cost of the route 3,2,0; however, because costs are rounded to the closest integer, the U-RR procedure was not able to recognize route 3,2,0 as the best route beyond this point.

Note further that the U-RR procedure alternates between routes that have the same rounded cost. More specifically, as the rounded cost estimates for the route 3,2,0 became equal to 3, the U-RR procedure chose the route that had the least number of unicast transmissions in order to improve its cost estimation, resulting in $r_{3,2,0}^{\left(URR\right)}\approx 0.5$. This behavior can be seen in Figure 9e: when node 5 generated between 3.75 and 7.5 packets/s, the U-RR procedure chose the route 3,1,0 more often than the LS-based procedure in order to improve the cost estimate of the route 3,1,0. This results in a slight increase in the number of node 3 retransmissions per 1000 messages of the U-RR procedure over the LS procedure, as can be seen in Figure 9f when node 5 generates around 4 packets/s.

It is also interesting to observe that it is possible for procedures to select non-optimal routes even when the estimation has low variation. For instance, when node 5 generated 0.5 messages/s $r_{3,2,0}^{\left(URR\right)}>0.9$, $\sigma_{3,2,0}^{\left(URR\right)}=0$, and ${\bar{c}}_{3,2,0}<{\bar{c}}_{3,1,0}$; however, the route 3,1,0 was still selected 10% of the time. This was probably due to node 3 missing RREQ messages from node 2 in one or more of the RREQ cycles.

#### 7.2.2. Scenario A2: WiFi Interference

**Figure 10:**
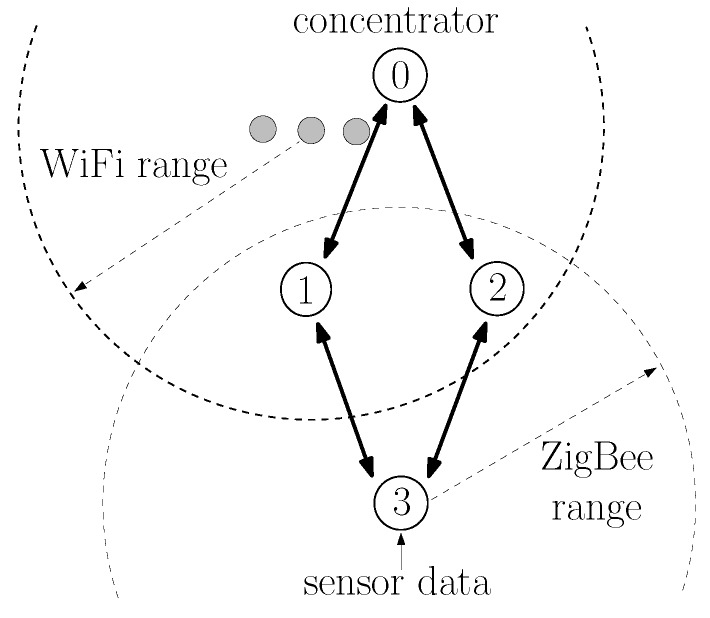
Topology where ZigBee devices share same spectrum as WiFi devices.

Consider the topology shown in Figure 10, where ZigBee nodes 0, 1, 2, and 3 operate in a communication channel that overlaps with the channel used by a WiFi IEEE 802.11n network. Consider that the ZigBee nodes are in the same locations as in the Scenario A1 of Section 7.1.2 and the WiFi network again consists of one access point and two stations, but now at locations (60,−30), (60,−20), and (60,−40) respectively. Assume that node 3 sends application data at an average rate of 20 packets/s to node 0.

At t=100 s, the WiFi access point starts two data streams of constant-bit-rate traffic of $R_{WiFi}$ bits/s, one to each WiFi station; and these streams last until the end of the simulation at t=400 s. Details of these streams are as described in Section 4. As in Scenario S2, the WiFi devices are far from node 3 and may transmit while node 3 is transmitting; however, because the WiFi devices are closer to node 1, the interference level increases at node 1 and decreases at node 2, resulting in $c_{3,1,0}>c_{3,2,0}$. In this case, it is desirable that node 3 chooses the route 3,2,0 as much as possible since node 2 is less subject to the WiFi interference.

**Figure 11:**
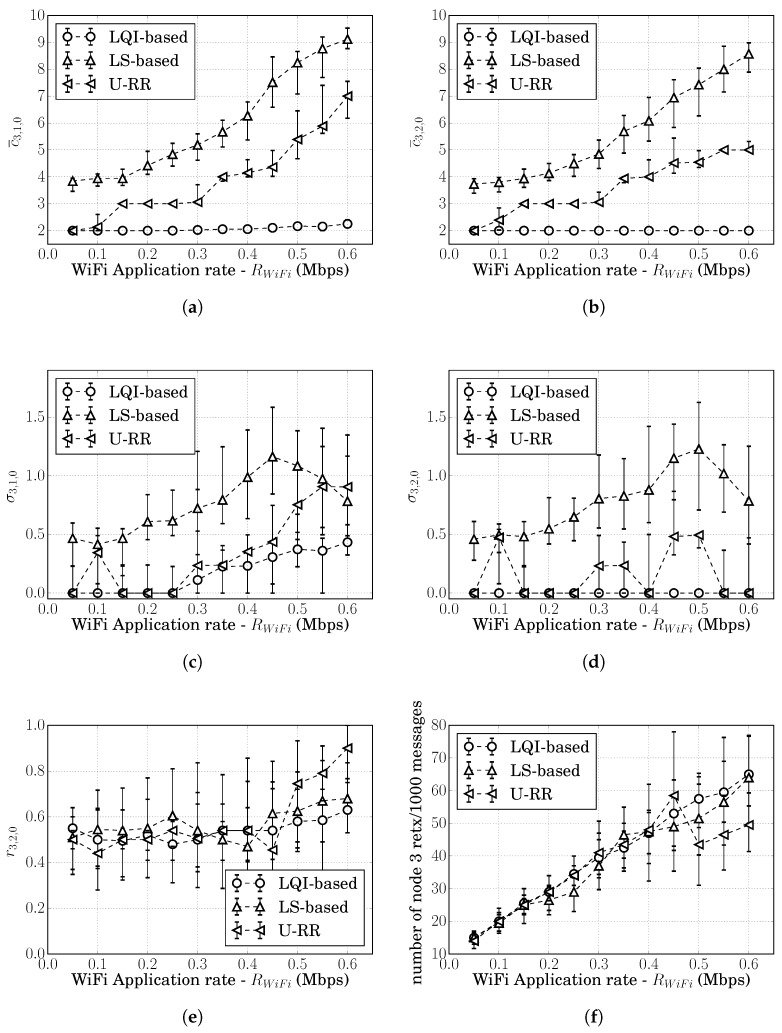
The median and the 15th to 85th percentiles of (**a**): the cumulative route costs of the route 3,1,0 (${\bar{c}}_{3,1,0}$), (**b**): the cumulative route costs of the route 3,2,0 (${\bar{c}}_{3,2,0}$), (**c**): the standard deviation of the estimated costs measured at node 3 for the route 3,1,0 ($\sigma_{3,1,0}$), (**d**): the standard deviation of the estimated costs measured at node 3 for the route 3,2,0 ($\sigma_{3,2,0}$), (**e**): the ratio of times that route 3,2,0 was chosen ($r_{3,2,0}$), and (**f**): the number of times that node 3 had to retransmit per 1000 messages generated in the scenario A2.

To analyze the performance of the U-RR procedure in selecting the best route 3,2,0, we varied the traffic level generated by the WiFi access point, and at each traffic level, we repeated the simulation 30 times, each time with a different random seed. Figure 11a through Figure 11f respectively, show the median and the 15th to 85th percentiles of the cumulative route costs 3,1,0 and 3,2,0 (${\bar{c}}_{3,1,0}$ and ${\bar{c}}_{3,2,0}$), their standard deviations ($\sigma_{3,1,0}$ and $\sigma_{3,2,0}$), the ratio of times that route 3,2,0 was chosen ($r_{3,2,0}$), and number of times that node 3 had to retransmit per 1000 messages generated for 30 simulation runs as WiFi traffic load $R_{WiFi}$ varied for the LS-based, the LQI-based, and the U-RR procedures.

Figure 11a,b show that, as the $R_{WiFi}$ increased above 450 kbps, both the LS-based and the U-RR procedures started to detect the higher cost of the route 3,1,0 with respect to route 3,2,0, while the LQI-based procedure still considered both routes having the same cost. It is interesting to observe that the ${\bar{c}}_{3,1,0}-{\bar{c}}_{3,2,0}\approx 0.5$ in the LS-based procedure, within the standard deviation of the estimates, while ${\bar{c}}_{3,1,0}-{\bar{c}}_{3,2,0}\approx 2$ in the U-RR procedure, showing that the U-RR procedure was able to better recognize route 3,2,0 as the best route. It is also interesting to observe that the standard deviations $\sigma_{3,1,0}$ and $\sigma_{3,2,0}$ of the LS-based procedure reduced for higher WiFi loads. This behavior can be explained by observing the cumulative route costs ${\bar{c}}_{3,1,0}$ and ${\bar{c}}_{3,2,0}$: when the WiFi load increased, there was a point in which the cumulative route costs increased above 8. Remembering that each individual path cost is quantized between 0 and 7, this suggests that $c_{1,0}$ and $c_{2,0}$ started to saturate at 7, reducing the variations in cost.

As shown in Figure 11e, all of the procedures struggled to recognize route 3,2,0 as the best route when $R_{WiFi}<400$ kbps; however, as $R_{WiFi}$ increased above 450 kbps, all of the procedures started to recognize route 3,2,0 as the best route, with the U-RR showing the best performance: when $R_{WiFi}=600$ kbps, both the LQI-based and the LS-based procedures were only sending a median of less than 70% of the packets through the route 3,2,0, while the U-RR based procedure sent a median of 90% of the packets through the route 3,2,0. As a result, as shown in Figure 11f, both the LQI-based and the LS-based procedures had more packet losses and retransmissions: while these procedures showed a median of 62 or more node 3 retransmissions/1000 messages generated, the U-RR procedure had an median of less than 50 retransmissions/1000 messages, representing a reduction of 20%.

#### 7.2.3. Scenario A3: Two-Hop Scenario

Consider again the topology of Figure 7 involving routes with more than 1 hop, with sensors at the same locations as described in Section 7.1.3.

In order to investigate the performance of the U-RR procedure when the scenario asymmetry is far from node 3, consider that node 3 generates an average of 20 packets/s; nodes 8 and 9 generate each an average of 0.5 packets/s; node 4 generates an average of 10 packets/s; and we vary the traffic load of node 5.

Because of the higher traffic load generated by node 4, the rate of interfering transmissions in node 1 increases, making the route 3,7,2,0 the best route in this scenario.

**Figure 12:**
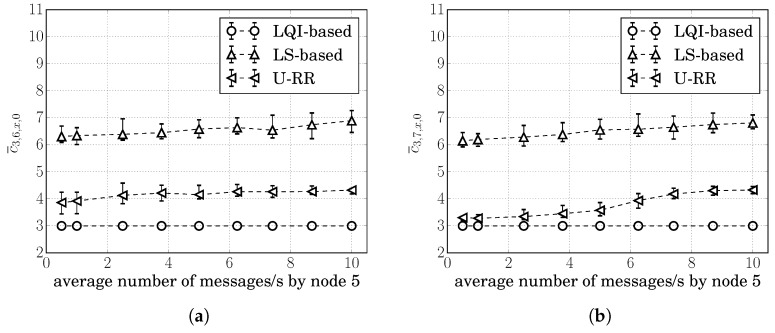

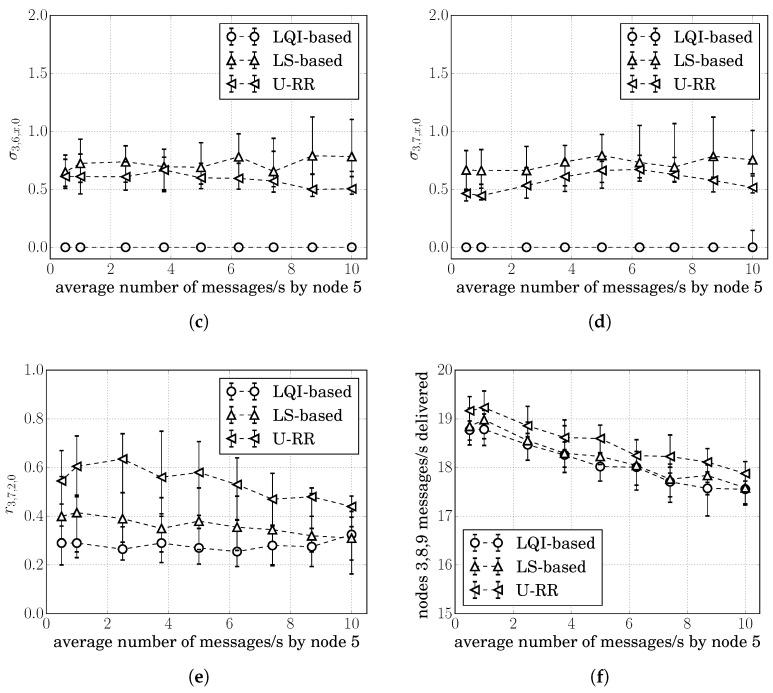
The median and the 15th to 85th percentiles of (**a**): the cumulative route cost of the routes 3,6,x,0 (${\bar{c}}_{3,6,x,0}$), (**b**): the cumulative route cost of the routes 3,7,x,0 (${\bar{c}}_{3,7,x,0}$), (**c**): the standard deviation of the estimated costs measured at node 3 for the routes 3,6,x,0 ($\sigma_{3,6,x,0}$), (**d**): the standard deviation of the estimated costs measured at node 3 for the routes 3,7,x,0 ($\sigma_{3,7,x,0}$), (**e**): the ratio of times that packets were relayed by the best routes 3,6,1,0 and 3,7,2,0 ($r_{3,6,1,0}+r_{3,7,2,0}$), and (**f**): the delivery rate of messages originated at nodes 3, 8, and 9 in the scenario A3.

Figure 12a,b show that, when node 5 generated 0.5 messages/s, both the LS-based and the U-RR procedures started to detect the higher cost of the routes passing through node 6 instead of node 7, while the LQI-based procedure was not able to differentiate routes 3,6,x,0 and 3,7,x,0. As before, the difference ${\bar{c}}_{3,1,0}-{\bar{c}}_{3,2,0}$ was higher in the U-RR procedure than in the LS-based procedure; however, in here, the difference was within the standard deviation of the estimates in both cases. Nevertheless, as shown in Figure 12c,d, the U-RR procedure showed a lower standard deviation of the cumulative costs when compared to the LS-based procedure.

As can be seen in Figure 12e,f, the U-RR procedure was able to choose the best route most often, and as a result, the U-RR procedure was able to deliver more messages from nodes 3, 8, and 9 that use nodes 6 and 7 as relays towards the concentrator. As the average number of messages/s generated by node 5 approached 10 packets/s, which is the same traffic loads generated by node 4, the costs of routes 3,7,2,0 and 3,6,1,0 became the same, and the U-RR procedure selected routes 3,7,2,0 and 3,6,1,0 with approximately the same frequency of around 45%.

### 7.3. Random Topologies

**Figure 13:**
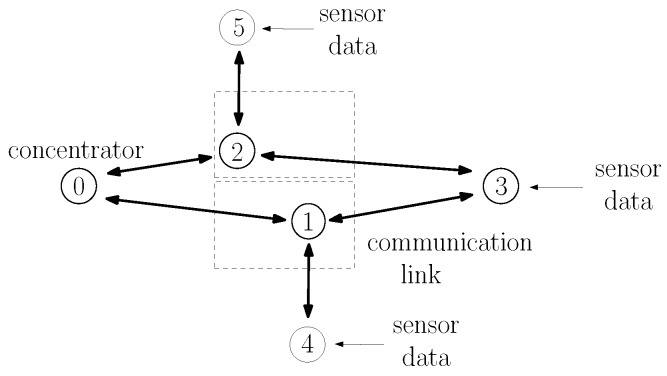
Topology where nodes 1, 2, 4, and 5 are randomly located.

Consider the scenario illustrated in Figure 13, where sensors at nodes 3, 4, and 5 send sensor measurements to the concentrator at node 0, and nodes 1 and 2 are available for routing packets. The difference between this and previous scenarios is that nodes 1, 2, 4, and 5 will at this stage be at random locations. More precisely, with node 0 located at (−80,0) and node 3 located at (80,0), node 1 will be uniformly distributed in the rectangular region with opposing vertices at (−10,−45) and (10,0); and node 2 will be uniformly distributed in the rectangular region with opposing vertices at (−10,45) and (10,0). The reason for placing nodes 1 and 2 at these rectangles is to create a scenario with 2 candidate routes; otherwise, if the random drops were such that only one route were available, all procedures would behave in the same way. Once nodes 1 and 2 are placed, as illustrated Figure 13, node 4 is placed 95 m below node 1 and node 5 is placed 95 m above node 2.

Assume that nodes 3, 4, and 5 respectively generate an average of 20, 10, 0.5 packet/s. Although node 5 generates less traffic load than node 4, route 3,2,0 is not necessarily the best route because the random position of nodes can cause different hidden node problems, and we compare the procedures based on the amount of retransmissions per 1000 messages.

We generated 30 sets of random locations for nodes 1 and 2, and for each set or random locations, we ran each of the three procedures using 30 different random simulation seeds.

**Figure 14:**
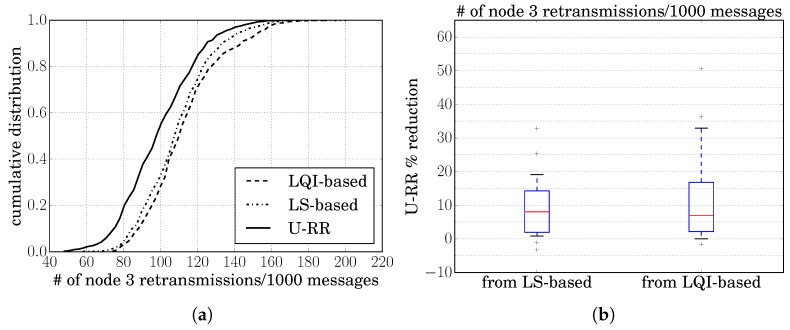
(**a**): Cumulative distribution of the number of node 3 retransmissions per 1000 messages generated. (**b**): Boxplots of the percentage reduction in the number of node 3 retransmissions per 1000 messages generated obtained by the U-RR procedure when compared to the LQI-based and the LS-based procedures.

Figure 14a shows the cumulative distribution of the number of node 3 retransmissions per 1000 messages generated by each procedure. It is possible to see that, with the U-RR procedure, 55% of the scenarios ran showed node 3 with 100 or less retransmissions per 1000 messages generated; and, with the LS-based or LQI-based, only 35% of the scenarios showed node 3 with 100 or less retransmissions per 1000 messages generated.

Figure 14b shows boxplots of the percentage reduction in the number of node 3 retransmissions per 1000 messages generated when compared to the LQI-based and the LS-based procedures. In each boxplot, the whiskers represent the 5th and 95th percentiles, the bottom and top parts of the box represent the 25th and 75th percentiles, and the line inside the box represents the median of the 30 random locations. When comparing against the LS-based and LQI-based procedures, the U-RR procedure reduced the amount of retransmissions by 14% or more in 25% of the random scenarios. In some scenarios, the reduction with respect to the LS-based procedure was 34%.

## 8. Conclusions and Avenues for Future Research

Although the three procedures offer similar performances at low traffic loads, this paper has shown that, at higher traffic loads, relying solely on link status (LS) messages or on the average of link quality indicators (LQI) of received packets to estimate link costs and select routes can degrade the performance of ZigBee’s route selection algorithm, particularly in asymmetric scenarios. This conclusion is in agreement with conclusions reached by other authors, which showed, in protocols other than ZigBee, that beacon-based link estimation procedures produce degraded performance.

Given the problems of LS-based and LQI-based procedures in higher traffic loads, we proposed the U-RR (unicast round-robin) procedure. The U-RR procedure uses MAC information regarding unicast transmissions to estimate the probability of successful transmissions on a link; and uses a modified route selection mechanism to decide among routes that have the same cumulative route cost. The modified route selection mechanism indirectly makes a node select among such routes in a round-robin fashion in order to improve the link cost estimation of candidate routes. The modified route selection mechanism is particularly important in ZigBee networks because ZigBee nodes have to quantize the link costs in just three bits, causing many route candidates to have the same cumulative route cost.

Our simulation results show that the U-RR procedure reduces the variance of link cost estimations, allowing nodes to better differentiate among routes, and increases the number of times that the best route is selected. The benefits of the U-RR procedure are more relevant under higher traffic loads and in asymmetric scenarios; however, even in other scenarios, the U-RR procedure performs at least as well as the LS-based procedure.

Lastly, it should be mentioned that the U-RR procedure is not necessarily the best link cost estimator and route selection procedure, and algorithms such as those mentioned in Section 3.1 would most likely offer improved performance; however, such algorithms would require a significant revision of the ZigBee protocol and frame formats. In contrast, our U-RR procedure offers improved performance to ZigBee networks while neither requiring new protocol messages nor changes to frame formats.

### Avenues for Future Research

Below are research areas that could complement the results presented here:

- Although this paper considered some scenarios involving WiFi interference, further scenarios involving WiFi interference would complement the results presented here. More simulations could also reduce the large variation observed in the scenarios with WiFi interference.
- The U-RR procedure proposed here considers a fixed observation window to estimate the cost of various routes. Since such an estimation depends on the number of unicast packets being transmitted, it would be interesting to study modifications in which the observation window adapts to the amount of unicast traffic generated.
- The U-RR procedure here was designed for the many-to-one routing algorithm of ZigBee. Although the ideas behind the U-RR procedure could also be applied in the other routing algorithms, new simulations and analysis would be necessary to determine whether the U-RR procedure would be useful in other routing algorithms as well.
- In this study, we considered the default parameters of the ZigBee network layer and the IEEE 802.15.4 MAC and physical layers. Given that the performance of these systems can vary with such parameters [29,30], it would be interesting to study whether the results reported here could be improved by optimizing such parameters.
- It would be interesting to study how the proposed route cost estimation procedure could operate together with clustering procedures [54,55,56,57,58]. Clustering procedures build a hierarchical topology in which sensors communicate with clusterheads, which forward the message to other clusterheads that relay the message until it reaches the destination. In the context of this paper, such clusterheads would be selecting routes towards the concentrator by using a route cost estimation procedure, such as the U-RR procedure, and it would be interesting to consider clustering algorithms that select clusterheads while taking into consideration the cost variations in the routes that interconnect them.

## Figures and Tables

**Table 1:** Mapping between the average link quality indicators (LQIs) of transmissions from a node $z_{1}$ and successfully received at node $z_{2}$ (${\bar{LQI}}_{z_{1},z_{2}}\left(t\right)$), and the estimated link cost ${\hat{c}}_{z_{1},z_{2}}^{\left(LQI\right)}\left(t\right)$.

Average LQI Interval	${\hat{c}}_{z_{1},z_{2}}^{\left(\mathrm{LQI}\right)}\left(t\right)$	Average LQI Interval	${\hat{c}}_{z_{1},z_{2}}^{\left(\mathrm{LQI}\right)}\left(t\right)$	Average LQI Interval	${\hat{c}}_{z_{1},z_{2}}^{\left(\mathrm{LQI}\right)}\left(t\right)$
$239\;<\;{\bar{LQI}}_{z_{1},z_{2}}\left(t\right)\;\leq \;255$	1	$185\;<\;{\bar{LQI}}_{z_{1},z_{2}}\left(t\right)\;\leq \;195$	4	${\bar{LQI}}_{z_{1},z_{2}}\left(t\right)\;\leq \;170$	7
$206\;<\;{\bar{LQI}}_{z_{1},z_{2}}\left(t\right)\;\leq \;239$	2	$174\;<\;{\bar{LQI}}_{z_{1},z_{2}}\left(t\right)\;\leq \;185$	5		
$195\;<\;{\bar{LQI}}_{z_{1},z_{2}}\left(t\right)\;\leq \;206$	3	$170\;<\;{\bar{LQI}}_{z_{1},z_{2}}\left(t\right)\;\leq \;174$	6		

**Table 2:** Mapping between the cost estimate ${\hat{c}}_{z_{1},z_{2}}^{\left(LS\right)}\left(t\right)$ received from node $z_{2}$ and the highest probability of successful transmissions that maps into ${\hat{c}}_{z_{1},z_{2}}^{\left(LS\right)}\left(t\right)$.

${\hat{c}}_{z_{1},z_{2}}^{\left(\mathrm{LS}\right)}\left(t\right)$	${\bar{p}}_{z_{1},z_{2}}^{\left(\mathrm{LS}\right)}\left(t\right)$	${\hat{c}}_{z_{1},z_{2}}^{\left(\mathrm{LS}\right)}\left(t\right)$	${\bar{p}}_{z_{1},z_{2}}^{\left(\mathrm{LS}\right)}\left(t\right)$	${\hat{c}}_{z_{1},z_{2}}^{\left(\mathrm{LS}\right)}\left(t\right)$	${\bar{p}}_{z_{1},z_{2}}^{\left(\mathrm{LS}\right)}\left(t\right)$	${\hat{c}}_{z_{1},z_{2}}^{\left(\mathrm{LS}\right)}\left(t\right)$	${\bar{p}}_{z_{1},z_{2}}^{\left(\mathrm{LS}\right)}\left(t\right)$
1	1.000	3	0.795	5	0.686	7	0.626
2	0.903	4	0.731	6	0.652		
